# Assessment of the Suitability of *Melilotus officinalis* for Phytoremediation of Soil Contaminated with Petroleum Hydrocarbons (TPH and PAH), Zn, Pb and Cd Based on Toxicological Tests

**DOI:** 10.3390/toxics9070148

**Published:** 2021-06-25

**Authors:** Teresa Steliga, Dorota Kluk

**Affiliations:** Department of Production Technology of Reservoir Fluids, Oil and Gas Institute—National Research Institute, Ul. Lubicz 25 A, 31-503 Krakow, Poland; kluk@inig.pl

**Keywords:** phytoremediation, *Melilotus officinalis*, soil, total petroleum hydrocarbons, polycyclic aromatic hydrocarbons, biodegradation, heavy metals, toxicological tests

## Abstract

The article presents issues related to the possibility of using toxicological tests as a tool to monitor the progress of soil treatment contaminated with petroleum substances (TPH, PAH), Zn, Pb and Cd in bio-phytoremediation processes. In order to reduce the high content of petroleum pollutants (TPH = 56,371 mg kg^−1^ dry mass, PAH = 139.3 mg kg^−1^ dry mass), the technology of stepwise soil treatment was applied, including basic bioremediation and inoculation with biopreparations based of indigenous non-pathogenic species of bacteria, fungi and yeasts. As a result of basic bioremediation in laboratory conditions (ex-situ method), the reduction of petroleum pollutants TPH by 33.9% and PAH by 9.5% was achieved. The introduction of inoculation with biopraparation-1 prepared on the basis of non-pathogenic species of indigenous bacteria made it possible to reduce the TPH content by 86.3%, PAH by 40.3%. The use of a biopreparation-1 enriched with indigenous non-pathogenic species of fungi and yeasts in the third series of inoculation increased to an increase in the degree of biodegradation of aliphatic hydrocarbons with long carbon chains and PAH by a further 28.9%. In the next stage of soil treatment after biodegradation processes, which was characterized by an increased content of heavy metals (Zn, Pb, Cd) and naphthalene, chrysene, benzo(a)anthracene and benzo(ghi)perylene belonging to polycyclic aromatic hydrocarbons, phytoremediation with the use of *Melilotus officinalis* was applied. After the six-month phytoremediation process, the following was achieved: Zn content by 25.1%, Pb by 27.9%, Cd by 23.2% and TPH by 42.2% and PAH by 49.9%. The rate of removal of individual groups of hydrocarbons was in the decreasing order: C_12_–C_18_ > C_6_–C_12_ > C_18_–C_25_ > C_25_–C_36_. PAHs tended to be removed in the following order: chrysene > naphthalene > benzo(a)anthracene > benzo(ghi)perylene. The TF and BCF coefficients were calculated to assess the capacity of *M. officinalis* to accumulate metal in tissues, uptake from soil and transfer from roots to shoots. The values of TF translocation coefficients were, respectively, for Zn (0.44), Pb (0.12), Cd (0.40). The calculated BCF concentration factors (BCF_roots_ > BCF_shoots_) show that heavy metals taken up by *M. officinalis* are mainly accumulated in the root tissues in the following order Zn > Pb > Cd, revealing a poor metal translocation from the root to the shoots. This process was carried out in laboratory conditions for a period of 6 months. The process of phytoremediation of contaminated soil using *M. officinalis* assisted with fertilization was monitored by means of toxicological tests: Microtox, Ostracodtoxkit F^TM^, MARA and Phytotoxkit^TM^. The performed phytotoxicity tests have indicated variable sensitivity of the tested plants on contaminants occurring in the studied soils, following the sequence: *Lepidium sativum* < *Sorghum saccharatum* < *Sinapis alba*. The sensitivity of toxicological tests was comparable and increased in the order: MARA < Ostracodtoxkit F^TM^ < Microtox. The results of the toxicological monitoring as a function of the time of soil treatment, together with chemical analyses determining the content of toxicants in soil and biomass *M. officinalis*, clearly confirmed the effectiveness of the applied concept of bioremediation of soils contaminated with zinc, lead and cadmium in the presence of petroleum hydrocarbons.

## 1. Introduction

Oil mining exploits valuable, non-renewable deposits of natural gas and oil but contributes to degradation of the natural environment. Works carried out in oil and gas mining are inevitably connected with generation and disposal of large quantities of waste materials, which in the years 1920–1950 were stored in excavation pits. The main pollution which hampers development and growth of flora on these sites are petroleum hydrocarbons (TPH, PAH) and heavy metals whose concentration in aged soils of mining pits is highly differentiated and has wide variations [1,2]. Petroleum pollution in soil provokes distortion in the course of natural cycles of circulation of matter and energy. One can observe stimulation of development of some groups of microorganisms and inhibition of activity of other ones. Filling soil pores makes exchange of air between the soil and the atmosphere difficult or impossible. A very large prevalence of organic carbon over nitrogen and phosphorus content entails acute deficiency of these components for microorganisms and plants. Degradation of properties of soil colloids (sorption capacity, ion exchange) occurs, provoking visible and detrimental changes in the environment. Moreover, some crude oil fractions can dissolve cell membranes and consequently disrupt the structure of the plant roots [3,4,5].

The high concentration of oil pollution (50–90 g kg^−1^ dry mass, performed within the laboratory using its own staff) in soil from weathered drill wastes causes major difficulties in bioremediation work [1,2,6]. Thus, it was assumed that the technological concept of soil treatment would be based on a stage-wise realisation of successive treatment processes which would allow a gradual decrease of petroleum pollution levels, which would in turn enable implementation of further methods of treatment of polluted sites. It encompasses basic bioremediation stimulated by bioventilation and enrichment of soil environment in biogenic components which facilitate development of indigenous microflora, as well as bioaugmentation consisting of inoculation of pre-cleaned soil with biopreparation based on indigenous bacteria, which in the final phase of the inoculation process were enriched in an isolated species of fungi [1,6,7].

Biodegradation of petroleum pollutions in soils, apart from the presence of microorganisms able to degrade polluters, also requires proper contents of nutrients, oxygenation, proper temperature conditions and pH reaction, suitable humidity and elimination of toxic compounds [8,9,10]. One of the main factors determining their vulnerability to biodegradation is bio-accessibility of the petroleum hydrocarbons and the chemical structure of hydrocarbons (length of chain, their branching, the presence of oxygen in hydrocarbon molecules, the presence and position of substituents, the structure and number of rings) [11,12]. Petroleum pollution constitutes a complex multi-component system, so the application of a mixture of bacteria cultures with an extensive enzymatic unit is recommended for its decomposition. In order to avoid an antagonistic reaction of microflora of indigenous soil to alien cultures of microorganisms not adapted to the environment, it is recommended to make bacteria consortia on the bases of indigenous microorganisms previously isolated from the soil [13,14,15]. The ability of microorganisms to adapt to pollutants plays a particularly essential role in the decomposition of xenobiotics. At the molecular level, adaptation is explained by a genetic variation of microorganisms (resulting from occurrence of spontaneous mutations and the presence of genetic elements) and then selection of these cells which have acquired the ability to use xenobiotics as sources of carbon and energy. The most active bacteria which possess the ability to biodegrade petroleum hydrocarbons (aliphatic and aromatic)—and the most often described in the literature—are the bacteria *Rhodococcus erythropolis*, *Micrococcus luteus*, *Pseudomonas putida*, *Pseudomonas fluorescens*, *Pseudomonas veronii*, *Rhizobium daejeonense*, *Mycobacterium friederiksbergense*, *Mycobacterium* *υanbaalenii*, *Nocardia asteroides*, *Sphingomonas paucimobilis* and *Sphingomonas yanoikuyae* [1,6,16,17,18,19,20,21]. Fungi also play a significant role in the removal of petroleum hydrocarbons through the production of intermediate products of quite often decreased toxicity and increased vulnerability to bacterial decomposition. That is why they should also be taken into consideration in the development of effective strategies of bioremediation. In the scientific literature, there have been presented many results the research into biodegradation processes with the use of such fungi as *Cladosporium, Aspergillus*, *Cunninghamella*, *Penicillium*, *Fusarium*, *Phanerochaete chrysosporium*, *Pleurotus ostreatus*, *Trichoderma asperellum* and *Cladophialophoria* [6,22,23].

The published reports indicate measurable effects of reclamation of degraded areas through the use of phytoremediation, which is an environmentally friendly and cost-effective remediation method which makes possible the removal of heavy metals and petroleum pollutants. (TPH, PAH) [24,25,26,27,28,29,30]. This method was used to decrease pollutants left in soil after the stage-wise process of biodegradation of petroleum pollutants was carried out.

The influence of phytoremediation plants on toxic compounds is variable, depending upon the properties of the pollution, types of plants and environmental conditions. In order to guarantee the possibility of phytoremediation, which is a slow process, plants with wide roots which grow quickly and tolerate pollution should be selected. The root is a key organ for stabilisation and accumulation of nutrients by a plant. Moreover, the root plays a key role in the adaptation of plants for their health and means of nutrition, due to such phenotypic traits as length of roots, biomass, density and area [31]. Pollutants, especially heavy metals, can change the permeability of the membrane in various species of plants, decrease enzymatic activity, distort nutrition of minerals, damage the photosynthetic apparatus and generate oxidative stress, influencing thus the morphology, increase and photosynthesis processes of plants. Excess heavy metals in cells induces molecular damage to plants mainly through the synthesis of reactive oxygen species (ROS) such as superoxide radical (O_2_^−•^), hydroxyl radical (^•^OH) and hydrogen peroxide (H_2_O_2_). Excessive synthesis of ROS is one of the initial responses to different stress factors in organisms. These ROS can lead to irreversible oxidization of lipids, proteins, chloroplastic pigments, DNA and RNA and thus affect cell viability [32].

The research carried out is showing that *Melilotus officinalis* can be effectively used for phytoremediation of soils polluted with heavy metals (copper, chromium, lead, molybdenum, zinc, nickel and cobalt) [26,33,34,35,36,37,38,39,40]. *Melilotus officinalis* is tolerant to soil contamination and can absorb more heavy metals during vegetation than other green plants. Vast research carried out both in laboratories and under in situ conditions has proven that *M. officinalis* is highly effective in the remediation of soils contaminated with organic pollutants, including TPH [41,42,43,44] and PAH [27,45,46,47].

The results obtained by Parrish et al. [27,46] imply a positive relationship between *M. officinalis* biomass development and PAH biodegradation. Much better results of degradation of PAH within the stretch 60–75% have been obtained after earlier application of biological treatment, as populations of microorganisms degrading PAHs showed 100-fold increases and the profile of the structural group of phospholipids of fatty acids (PLFA) changed during the increase, indicating a change in the system structure.

The investigation of phytoremediation carried out until now has focused mainly on one type of pollution—there is no information on the interaction of heavy metals and co-existing petroleum pollutants (TPH and PAH) during their accumulation and transport in plants, particularly in *M. officinalis*. However, plants which are able to clean soil contaminated with heavy metals in the presence of petroleum substances seem to have a huge potential which can be widely used to restore use and value to contaminated areas.

Selection of suitable monitoring plays an essential role in the assessment of effectiveness of biodegradation and phytoremediation processes of soils contaminated with heavy metals and petroleum substances. The literature shows a limited amount of information relating to the possibility of applying toxicological monitoring to soils polluted with heavy metals and petroleum products as a tool helpful in assessing the progress of remediation of polluted areas. An objective assessment of the degree of hazard posed by polluted areas is given by toxicological examinations, which consist of observation of morphological changes of bio-indicators in contact with xenobiotics and their metabolites. Bio-indicators usually used in bio-tests belong in principle to main groups of organisms, such as bacteria, invertebrates and more complex plants. In the toxicological monitoring of remediated soils, a set of tests have been used which comprised the following tests: Microtox^®^ Solid Phase SPT [48,49] test of direct contact Ostracodtoxkit F™ [6,50], phytotoxicity test Phytotoxkit F™ [51,52,53] and microbiological assay for risk assessment test (MARA) [54,55,56]. These tests use organisms which are dormant or inactivated (forms of crypto-biotics) and which—after termination of the whole procedure—can be used to determine the degree of toxicity (e.g., of soils).

We hypothesized that it is possible to apply a set of technologies that apply the gradual reduction of petroleum pollutions in biological processes in combination with phytoremediation using *Melilotus officinalis* for the treatment of soil contaminated with co-occurring petroleum hydrocarbons (TPH and PAH) and heavy metals (Pb, Cd, Zn) and the use of toxicological examinations aimed at determining changes in toxicity during biodegradation and phytoremediation processes. The aim of the research study was: (a) determination of the effectiveness of the biodegradation process of petroleum pollutants during basic bioremediation stimulated by enrichment of the soil environment with biogenic compounds and inoculation of soil with bio compounds made on the basis of indigenous non-pathogenic species of bacteria, fungi and yeast; (b) determination of the effectiveness of the phytoremediation process, assisted by soil fertilization of soil contaminated with heavy metals and petroleum hydrocarbons (TPHs and PAHs) left after initial biodegradation processes; (c) determining the impact of the presence of TPH and PAH on the process of soil purification from heavy metals and (d) toxicological monitoring of soil function during biological and phytoremediation processes of soil, together with sensitivity assessment of the applied biotests. For this purpose, a package of state-of-the-art toxicological examinations has been used with bioindicators belonging to various taxonomic groups (bacteria, crustaceans and more developed plants), representing all trophic groups: producers, consumers and reducers.

## 2. Materials and Methods

### 2.1. Soil Description

Soil samples were taken from weathered drill wastes G-6 situated in the south-eastern part of Poland (22°4′42.99″ E, 49°40′16.5″ N) (Soil B), which contained soil mixed with drilling wastes. Soil samples were also collected from a forested area in the vicinity of the weathered drill wastes (22°4′42.2″ E 49°40′17.15″ N). This soil served as a “background” (Soil A). All 30 soil samples were taken from the surface layer (0–0.30 m below the ground level) of each site. Each type of soil was mixed in order to average it. The samples prepared in this way were subject to chemical and physical analyses, the results of which are presented in Table S1.

The analyses of petroleum pollutants (TPH and PAH) showed their high concentration in the soil B (TPH = 56,371 mg kg^−1^ dry mass PAHs = 139.28 mg kg^−1^ dry mass). In order to reduce the level of weathered petroleum pollutants in soil B, the technology of step-wise treatment was used, including basic bioremediation (Soil B1) and inoculation with biopreparation-1 based on indigenous microorganisms, which was carried out in ex-situ using the prism method (1st series 40 days—Soil B2, the 2nd series 45 days—Soil B3) and then biopreparation-2 from indigenous bacteria enriched in fungi and yeast (3rd series 50 days–Soil B4). After the process of biodegradation of petroleum pollutants, Soil B4 was used as investigated material in the phytoremediation processes, aiming at a decrease in heavy metal content (Zn, Pb, Cd) in the presence of petroleum pollutants (TPH and PAH). For comparison, the phytoremediation process of uncontaminated soil (Soil A) was carried out.

### 2.2. Biopreparation Characteristics

The scope of microbiological tests aimed at developing a professional biopreparation based on bacteria and fungi from the soil of the G-6 (Soil B) pit was extended to include the use of modern molecular techniques used to determine the diversity of the population of microorganisms in bioremediation processes [2,6,8].

As a result of these research, 22 bacterial strains were isolated, 2 strains of fungi and 1 strain of yeast. From all isolated microorganisms the major part consisted of representatives of the biological row *Acetinomycetales* (type *Acetinobacteria*). These microorganisms are frequently found in soils and characterised by their ability to decompose petroleum hydrocarbons. The strain *Rhodococcus* was particularly common. The characteristic feature of isolated strains was their ability to use not only aliphatic hydrocarbons but also aromatic compounds, although common ways of utilising these hydrocarbons were not frequent. Petroleum hydrocarbons were present in the soil in the weathered drill wastes for decades, and it was possible that microorganisms which survived in this hostile environment orientated their metabolism towards decomposition of compounds found in the petroleum pollutants.

In order to identify more precisely microorganisms contained in the prepared biopreparations, we carried out chain reaction of polymerase PCR and analysis of coding sequences of the gene 16S rRNA in bacteria and 18S rRNA in fungi. The obtained results were compared with the GenBank database using the BLAST program. These tests constituted the basis for proper characterisation of species of microorganisms isolated from Soil B [6,57].

For the purpose of biodegradation of petroleum pollutants, biopreparation-1 was prepared on the basis of indigenous bacteria, which was later modified by isolated species of fungi and yeast (biopreparation-2) (Table S2).

While preparing a professional biopreparations, particular attention was paid to safety of its use. Molecular examination allowed for the determination of species affiliation to isolated and selected microorganisms which belonged to pathogenic species. It was based on the list of classification used by the American Type Culture Collection (ATCC) (Biosafety Level-1—where no cases of causing any diseases among healthy, adult persons have been observed). Description microbiological analysis provided in the Supplementary Materials.

### 2.3. Biodegradation of Petroleum Pollutants in Ex-Situ Conditions

Tests on the biodegradation of TPH and PAHs on the soil from weathered drill wastes G-6 (Soil B) was carried out means of the ex-situ prism method. The soil at an amount of 50 kg was placed on a specially designed test stand, ensuring constant temperature (20–25 °C) and humidity (20–25%) during the experiment (Supplemental Figures S1 and S2). The task outlined for basic bioremediation (1st stage of purification—Soil B1) was to increase the amount of indigenous microorganisms through enrichment of the environment in nutrients and optimization of the process parameters, here for proper activity of microorganisms, the C:N:P ratio should be approx. 100:10:1 [8,9]. However, because of various speeds of decomposition of hydrocarbons and the unknown degree of assimilation of nutrients by microorganisms (depending upon the type of soil), optimal doses of mineral fertilizers were found in laboratory tests (N:P = 8:1). In the research of basic bioremediation carried out over 40 days, biogenic substances were gradually dosed (nitrogen and phosphorus) in the form of the mineral fertilizer “Azofoska”, composed of 13.6% total nitrogen, 5.5% nitrate nitrogen, 8.1% ammonia nitrogen, 6.4% soluble P_2_O_5_, 19.1% K_2_O in the form of K_2_SO_4_, 4.4% MgO in the form of MgSO_4_ and microelements (0.17% Fe, 0.27% Mn, 0.18% Cu, 0.045% Zn and 0.09% Mo) to obtain optimum proportions. These values were determined on the basis of soil parameters monitoring; dehydrogenase activity and a decrease in the content of petroleum pollutants in the tested soil. Similar methods of selecting biogenic substances were used by other scientists in their research [6,7,8,9,58]. The soil reaction of Soil B was corrected by supplementing agricultural limestone in the amount 1.0–1.5 g kg^−1^ of soil till obtaining the optimum pH 7.5–7.8.

The inoculation was carried out using biopreparation-1 based on indigenous bacteria with a density of 10^8^–10^9^ cfu ml^−1^ in two series (1st series 40 days—Soil B2, the 2nd series 45 days—Soil B3) and then biopreparation-2 from indigenous bacteria enriched in fungi and yeast (3rd series 50 days–Soil B4).

The course of the biodegradation process of petroleum pollutants was controlled by monitoring defined physical and chemical parameters, with special attention to chromatographic analyses and toxicological tests. Soil B4, following these biodegradation processes, served as reference material in the process of phytoremediation whose aim was to remove the remaining petroleum pollutants (TPH and PAH) and heavy metals (Pb, Zn, Cd).

### 2.4. Plant Selection, Experiment Description

*Melilotus officinalis*, known also as yellow sweet clover, is a leguminous plant with a deep system of taproots and the ability to bond with nitrogen from the atmospheric air. Due to a high ability to adapt to a wide variety of soil conditions, *M. officinalis* can grow in nutrient-poor soils and also in drought conditions. These species have a symbiotic relationship with the bacteria *Rhizobium*, what allows the species *Melilotus* to grow in nitrogen-poor soils. This plant creates extensive root systems and shows the ability of phytoremediation of moderately salinated soils [36,38,46,59,60]. Roots of *M. officinalis* produce quite significant amounts of cumarin, which stimulates co-metabolism of pollutants, including polychlorinated biphenyls (PCB) and PAH [61]. This plant was selected for examination of effectiveness of the phytoremediation process of soils polluted with metals Zn, Pb, Cd and petroleum substances (TPH and PAH).

The tests were carried out for 6 months under laboratory conditions in an established pot culture with *M. officinalis* in a soil containing known toxicant doses. In order to provide advantageous conditions for growth of plants during the phytoremediation process for selected soils, there were introduced fixed doses, determined on the basis of laboratory tests of the agricultural limestone and mineral fertilizer “Azofoska”.

In the results of the fertilizing process carried out in all types of soil (i.e., Soil AF and Soil B4F), the optimal ratio of nitrogen and phosphorus was found (N:P = 10:1) and pH value was within the limits 7.6–7.8. The experiment was carried out in two series of pot tests in the examined soils (Soil A and Soil B4) without cultivation and in soils with cultivation of *M. officinalis*. For this purpose, about 2000 g of soil were taken twice and placed in experimental pots of 15 cm diameter and 20 cm height. In two double pots containing soil AF and soil B4F there was planted *M. officinalis*. The remaining two pots, containing investigated soil without plants, served as reference soil. The process of phytoremediation of contaminated soils was accompanied by monitoring of their composition with regards to the concentration of Zn, Pb and Cd as well as TPH and PAH. Isolation of pollutants from the soil samples was carried out in the initial phase of the experiment and then after 2, 4 and 6 months. Moreover, after termination of the phytoremediation process, which lasted for 6 months, the collected plants were washed with demineralized water and divided into roots and shoots. The obtained biomass was dried and weighed. The plant materials (shoots and roots) were mineralized, and the content of Zn, Pb and Cd was determined.

#### 2.4.1. Physical and Chemical Tests of Soil and Biomass

In the soil prepared for experiment, humidity was measured by way of determining mass decrement, as an effect of evaporation of water during drying in a temperature of 105 °C. The rest of the soil was brought to the airdried state and passed through a sieve with 1-mm mesh size. Then, physical and chemical analyses were performed after extraction of analytes.

After this process of purification was carried out, the soil was characterized by elevated content of heavy metals (Zn, Pb, Cd), and TPH, as well as naphthalene, chrysene, benzo(a)anthracene and benzo(ghi)perylene, belongs to the group of PAH (Table S1).

#### 2.4.2. Extraction of Analytes from Soil and Biomass, and Methods Used to Determine the Analytes

The analytes being determined were extracted from the soil matrix through extraction of organic substances from soil with dichloromethane in a Soxhlet extractor, extraction of substances soluble in water (soil-to-water weight ratio = 1:10), soil mineralization in concentrated, spectrally pure HNO_3_ solution in a microwave mineralizer, Magnum II.

The water extract of the soil was analysed to determine its physical and chemical parameters, including pH reaction, conductivity and content of cations: K^+^, NH_4_^+^ and anions: F^−^, Cl^−^, NO_3_^−^, PO_4_^3−^ and SO_4_^2−^. Soil components extracted through mineralization were analysed to determine the content of Ca, Mg, Si, Al and heavy metals (As, Cd, Cr, Co, Cu, Pb, Mo, Ni, Sn, Zn). Plant material collected during the experiment was weighed, dried and mineralized, and then analysed to determine Zn, Pb and Cd content. A 1-g sample of each part of the plant material (shoot and root) was mineralized.

Water solution reaction for soils was determined using a potentiometric method, while anions and cations were determined by an adequate chromatographic methodology using an ion chromatograph from Sycam. Before any new analytical method was initiated, the chromatograph was configured by proper connection of columns and detectors. Each tested application of ion determination required calibration curves to be prepared. The curves were prepared using certified standard solutions from Spectracer.

The LCA A14 column was used for chromatographic determination of anions, and 5 mM Na_2_CO_3_ solution with modifier (4-hydroxybenzonitrile) was used as an eluent. Suppressed conductivity detection was used during the determination process. The alkali metals and ionic NH_4_^+^ were separated in the LCA K016 column; mixtures of 4 mM HNO_3_: CH_3_OH solution (7:3) were used as the eluent, and determination was carried out using conductometric detection with reverse function. Heavy metal cations were separated in the LCA K02 column using 0.1 M tartaric acid, and then derivatized with 4-(2-pyridyl-(2)-azo)resorcinol (PAR) and detected using the UV/Vis detection [62,63].

Petroleum pollutants (TPH) were determined by means of extraction with dichloromethane (POCH S.A., Poland), which was carried out in three series (20 mL of solvent, 15 min) by sonification in an ultrasonic bath (Sonoswiss SW 6H) of ultrasound frequency 30 kHz. Florisil solid-phase extraction cartridges (no. 7213-03) were evaluated for cleanup of polar substances. The solvent was evaporated in rotary vacuum evaporators, and the extract was dissolved in 1 mL of dichloromethane and analysed by the GC method. In order to determine efficiency of extraction with recovery at the level of 95.9%, substitute standard o-tertfphenyl was used. The analysis of petroleum hydrocarbons contained in the tested soil, including the identification of n-alkanes (n-C_6_–n-C_40_) and volume determination of total petroleum hydrocarbon content (TPH), was carried out using the Clarus 500 GC chromatograph from Perkin Elmer (Table 1). As a bio-marker certified benchmark C30 17α(H), 21β(H)-hopan (Supelco, Bellefonte, PA, USA) no. 08189 was used [6,7].

Determination of PAH comprised isolation of analytes PAH by means of continuous extraction (Soxhlet) with the use of petroleum benzin at 40–60 °C; separation of the fraction PAH with the use of two-phase cartridges Bakerbond SPE PAH Soil, containing phases CN/SiOH, elution of analytes with the use of mixture of solvents acetone, toluene = 3:1. The content of PAH was determined by means of gas chromatography, the parameters of which are presented in Table 1.

#### 2.4.3. Calculation of Phytoremediation Process Parameters

The following parameters were analysed to assess the effectiveness of the soil purification process through phytoremediation carried out using *Melilotus officinalis*: (a) plant biomass (dry mass of roots and shoots), (b) total metal concentration in plant tissues (roots and shoots), (c) translocation factors (TFs):TF = C_shoot_/C_root_, (d) bioconcentration factors (BCFs): BCF_plant_ = C_plant_:C_soil_, where C_plant_ and C_soil_ are metal concentration in a plant (shoot and root) and in soil, respectively. Metal concentrations in roots, shoots and soil are given in (mg kg^−1^) [61,64].

### 2.5. Toxicological Tests of Soil

Apart from determining toxicant concentration, the effectiveness of applied phytoremediation procedures in soil purification process was assessed based on toxicological test results. Soil samples were analysed using 4 microbiotests: (Phytotoxkit^TM^) [51,52,53,56], Ostracodtoxkit F^TM^ [6,50], (Microtox^®^STP) [48,49,51] and MARA [54,55,56,65]. The tests were performed according to standard procedures.

Material for testing consisted of control soil and soil after fertilization subject to the phytoremediation process. Toxicological tests were performed before starting the phytoremediation process (soils AF, B4F), after 2 months (soils AFPh-2, B4FPh-2) or 4 months (soils AFPh-4, B4FPh-4), and after the conclusion of the 6-month experiment (soils AFPh-6, B4FPh-6).

#### 2.5.1. Phytotoxkit^TM^

Chronic toxicity assessment test Phytotoxkit^TM^ is based on the evaluation of germination and early growth of plants (root elongation inhibition measurement), which is standardized as per Polish standard [PN-ISO 11269-1:1998]. Three types of plants selected according to germination rate and root growth rate are used in the test, which allows making a complete determination in 3 days of incubation: monocotyledonous—sorghum (*Sorghum saccharatum*), and dicotyledonous—cress (*Lepidium sativum*) and white mustard (*Sinapis alba*). The determination process was carried out using three repetitions for each test plant. The tests were performed on transparent polystyrene test plates. Incubation conditions: temperature T = 25 °C in darkness, incubation time t = 72 h. Test reaction: inhibition of germination and early growth of the root.

#### 2.5.2. Ostracodtoxkit F^TM^

Chronic toxicity assessment test Ostracodtoxkit F^TM^ uses young demersal crustaceans *Heterocypris incongruens* hatched from spore cysts in 52 h. Their percent death rate and growth inhibition are determined after 6 days of contact with examined sediment with reference to the results obtained in contact with nontoxic control sediment. The test is performed on plates with 6 holes (3 × 2), incubation time: 6 days at the temperature.

#### 2.5.3. Microtox^®^/DeltaTox

Test Microtox^®^ Solid Phase—acute toxicity test based on measurement of fluorescence for *Vibrio fischeri* bacteria, which, under normal conditions, use ca. 10% of their metabolism on the production of light. In the electron transport system of these bacteria, the luciferase enzyme (alkane oxygenase) catalyses oxidation of the reduced substrate (reduced flavin mononucleotide, riboflavin phosphate or flavin-adenine dinucleotide). Luminescence, which is measurable with a photometer, takes place during this process. Generated substrates involved in this reaction are oxygen and long-chain aldehydes. In the presence of substances negatively affecting cellular metabolism, bacteria react instantly with a drop in their luminescence, which is measured using the Delta Tox analyser after a 15-min contact with the sample. Toxicity results were calculated as EC50—tested sample concentration triggering a 50% test survival reaction (PE).

#### 2.5.4. MARA

MARA (Microbial Assay for Risk Assessment) is an innovative environmental risk assessment test in which the system for assessing chronic toxicity of samples uses ten prokaryotic organisms—bacteria of different taxonomy and one eukaryotic organism—yeast as bioindicators. Strains used in the MARA test include: *Microbacterium spaciec*, *Brevundimonas diminuta*, *Citrobacter freudii*, *Comamonas testosteroni*, *Entrococcus casseliflavus*, *Delftia acidovorans*, *Kurthia gibsoni*, *Staphylococcus warneri*, *Pseudomonas aurantiaca*, *Serratia rudidaea* and *Pichia anomala*.

Lyophilized bioindicators are placed by the manufacturer in the cells of the polystyrene 96-well microplate, which is then hermetically packaged under aseptic conditions. In the MARA test, the toxicity of the sample is evaluated based on the degree of inhibition of growth of the test organisms after 18 h of incubation. An observed visual effect is the water-insoluble red dye pellets produced by healthy bacteria (reduction of a tetrazolium red). After the test, the plate is scanned, and its image is analysed by special image analysis software.

### 2.6. Statistical Analysis

Statistical analysis of obtained chemical, chromatographic and toxicological test results was carried out using analytical and graphical methods. One-way analysis of variance (ANOVA) was completed, and then, Tukey’s post hoc test made it possible to eliminate statistically insignificant analytical data at the confidence level *p* < 0.05. The “Statistica” application, ver.13.1 for Windows, was used to process statistically obtained analytical data.

## 3. Results

### 3.1. Soil Tests

The pH values of water extracts from tested soils (10:1) are diverse and range from 6.1 to 6.5 (Supplemental Table S1). The soil analysis accomplished to determine nitrogen and phosphorus compound content showed that the amount of these components did not provide optimal conditions for the development of microbiological flora. In order to correct these parameters, the soil was fertilised with additional mineral fertilizer and agricultural limestone, attaining thus optimal values (N:P = 10:1 and pH = 7.6–7.8) advantageous for useful microorganisms. The investigated soils showed low values of conductivity of water extract (133–204∙μS cm^−1^) and COD (62–224 mg O_2_∙dm^−3^). Humidity of the investigated soil, which is of the utmost significance for bioavailability of metals and biogenic substances in rhizosphere, evolved within the range of 38.4–62.4%. The investigated soils contained small quantities of chlorides and sulphates. A higher concentration of heavy metals was found in Soil B and Soil B4 compared to Soil A, where zinc reached levels of 375.8 and 395.2 mg∙kg^−1^ dry mass, lead 285.6 and 264.4 mg∙kg^−1^ dry mass and cadmium 11.0 and 9.5 mg∙kg^−1^ dry mass.

The soils investigated in the experiment had variable content of petroleum hydrocarbons (TPH), which were found within the values 502.3–56,371 mg∙kg^−1^ dry mass. Significantly higher concentration of these components was found in the soil from the cleaned weathered drill wastes (Soil B), where hydrocarbons of the chain length C_10_–C_22_ prevailed (63.7%). In the soil subject to the phytoremediation process (Soil B4), pristan (9.8%) and fitan (7.7%) dominated, and there were hydrocarbons with carbon chains of length C_9_–C_13_ (10.1%) and C_24_–C_36_ (31.4%).

PAH content in the investigated soils evolved at the level 0.73–139.28 mg∙kg^−1^ dry mass; most of them were found in Soil B. In this soil, the following hydrocarbons dominated: naphthalene (58.7%), benzo(a)anthracene (16.0%), chrysene (10.5%) and benzo(ghi)perylene (7.5%). The stage-wise bioremediation process, carried out ex situ, caused considerable decrease in PAH content in Soil B to the level of 26.8 mg kg^−1^ dry mass (Soil B4), while the highest values are as follows: naphthalene (13.56 mg kg^−1^ dry mass), benzo(a)anthracene (4.56 mg kg^−1^ dry mass), chrysene (2.76 mg kg^−1^ dry mass) and benzo(ghi)perylene (3.08 mg kg^−1^ dry mass).

From the presented results of chemical determination, it can be stated that Soil A, taken from the forested area in the vicinity of the weathered drill wastes G-6, is not polluted, whereas the soil taken from the surface layer of the mining pit which was tested ex-situ by the stage-wise purification process (Soil B4) shows higher values of Zn, Cd, Zn and TPH, as well as naphthalene, chrysene, benzo(a)anthracene and benzo(ghi)perylene from the group of polycyclic aromatic hydrocarbons, and it is recommended to remediate it.

### 3.2. Evaluation of the Biodegradation Efficiency of TPH and PAH

#### 3.2.1. Evaluation of the Biodegradation of TPH and PAH Based on Chromatographic Analyses

**Figure 1:**
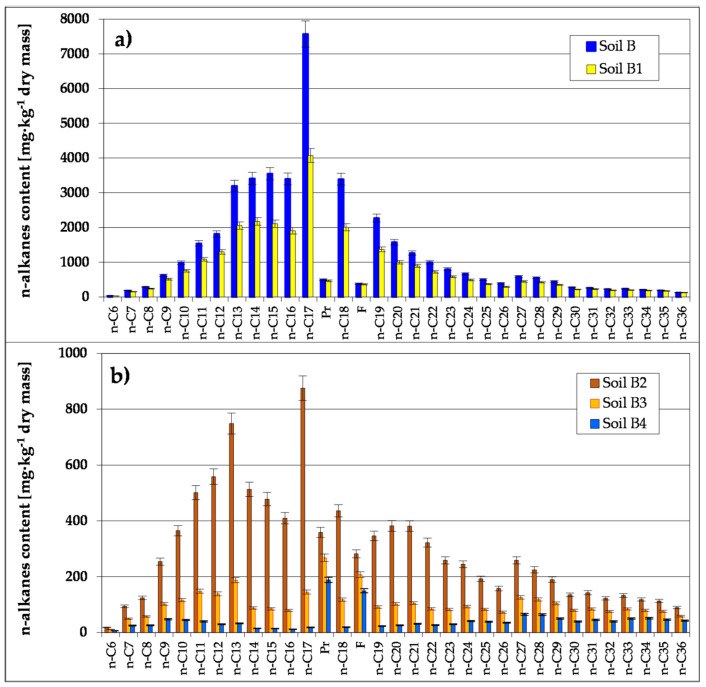
Comparison of identified n-alkane contents in Soil B: (**a**) after basic bioremediation; (**b**) after successive series of inoculations in laboratory conditions—ex-situ method (repetition number *n* = 9–10 *p* < 0.05). (Soil B) raw soil; (Soil B1) soil after basic bioremediation; (Soil B2) soil after inoculation with I biopreparation (1st series); (Soil B3) soil after inoculation with I biopreparation (2nd series); (Soil B4) soil after inoculation with II biopreparation (3rd series).

**Figure 2:**
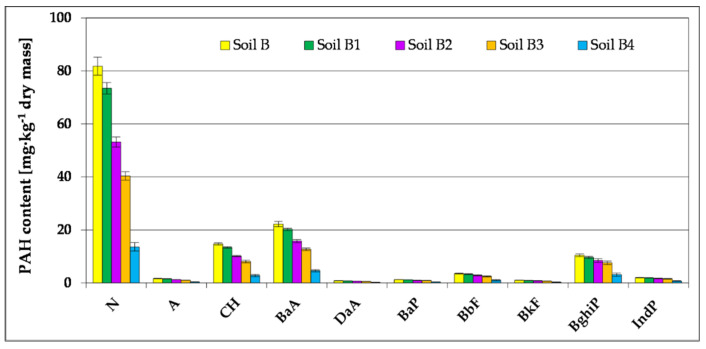
Alternation in polycyclic aromatic hydrocarbons (PAH) contents after consecutive stages of soil B purification in laboratory conditions—ex-situ method. (Soil B) raw soil; (Soil B1) soil after basic bioremediation; (Soil B2) soil after inoculation with I biopreparation (1st series); (Soil B3) soil after inoculation with I biopreparation (2nd series); (Soil B4) soil after inoculation with II biopreparation (3rd series). Naphthalene (N), Anthracene (A), Chryzene (CH), Benzo(a)anthracene (BaA), Dibenzo(a,h)anthracene (DaA), Benzo(a)pyrene (BaP), Benzo(b)fluoranthene (BbF), Benzo(k)fluoranthene (BkF), Benzo(ghi)perylene (BghiP), Indeno(1,2,3-cd)pyrene (IndP).

Biodegradation of petroleum pollutants in Soil B by means of stage-wise technology was carried out using the prism method in ex-situ conditions—which comprised basic bioremediation and inoculation (accomplished in three series with biopreparations prepared on the basis of indigenous bacteria, fungi and yeast (Table S2)—resulted in a considerable reduction of petroleum pollutants (TPH) went from 56,371 to 1932-mg kg^−1^ dry mass, and polycyclic aromatic hydrocarbons from 139.28 to 26.80 mg kg^−1^ dry mass. The whole cycle of soil purification was controlled by means of chromatographic determination of petroleum pollutants. The chromatographic analyses allowed for assessment of the degree of biodegradation of particular petroleum hydrocarbons (n-alkanes), which form part of TPH pollutants (Figure 1 and Figure 2, Supplemental Tables S3 and S4). Many reports have revealed that the best method of treating hydrocarbon degradation in soil contaminated with petroleum substances is a combination of biostimulation and bioaugmentation [15,17,66].

The presented results of chromatographic analyses allow for the opinion that reduction in the content of petroleum pollutants was reached during basic remediation, from 56,371 to 37,220 mg TPH kg^−1^ dry mass (Soil B1). The highest degree of reduction of oil hydrocarbon pollutants was noted for hydrocarbons of the aliphatic series, within the following values: for n-C_13_–n-C_22_ (35.7–46.2%) and for n-C_21_–n-C_29_ (21.7–29.3%), while for heavy hydrocarbons n-C_31_–n-C_36_ it was lower and ranged from 3.9 to 15.0% (Figure 1).

During the basic bioremediation process, a decrease in the content of polycyclic aromatic hydrocarbons from 139.28 to 126.16 mg kg^−1^ dry mass (Soil B1) was also noted. The highest degree of reduction in the content was recorded for 2-ring naphthalene, which was 10.2%. A slightly lower degree of biodegradation was achieved for PAH of the 3-ring anthrancene (A), at the level of 7.3%. On the other hand, for 4-ring PAH (BaA, CH) the degree of their reduction ranged from 8.8 to 9.01%. 5-ring PAH (BbF, BkF, BaP, DaA) were much more difficult to biodegrade, as the effectiveness of the primary bioremediation process was determined at the level of 5.0–8.0%. In contrast, PAH containing 6 rings per molecule (IndP and BghiP) are biodegradable at a very low level: 4.1–7.7% (Figure 2).

Chromatographic analysis proved that during inoculation with the biopreparation-1 (1st series) within 40 days, the concentration of TPH is reduced from 37,220 to 14,041 mg kg^−1^ dry mass. The quickest biodegradation process takes place with aliphatic hydrocarbons with the carbon chain of the length n-C_11_–n-C_23_, at the level of 55.6–78.5%. Hydrocarbons with carbon chain length n-C_24_–n-C_36_ were also biodegraded in satisfactory degree, within the limits 28.3–50.3% (Soil B2). These results prove that the biopreparation-1 used in the first series was effective. Due to the slowing biodegradation process of n-alkanes in the final stage of inoculation, the testing was continued in the second series of inoculation. The second stage of inoculation, carried out for 45 days with the use of biopreparation-1, made possible the reduction of TPH from 14,041 to 5043 mg kg^−1^ dry mass. The highest level of reduction of aliphatic hydrocarbon content was noticed for n-alkanes with length of carbon chain n-C_11_–n-C_22_, in the range of 70.5–83.4%. The lowest degree of biodegradation was for hydrocarbons with the carbon length n-C_23_–n-C_36_, whose content in the remediated soil was reduced by 34.4–68.2% (Soil B3).

Carrying out in two series inoculation of soil with biopreparation-1 on the basis of non-pathogenic species of bacteria contributed to reduction of PAH content within 85 days from 126.16 to 75.68 mg kg^−1^ dry mass. (Soil B3). The reduction of particular identified polycyclic aromatic hydrocarbons in the successive two series of inoculation was comparable. The highest degree of reduction was observed in the case of 2-cycle naphthalene (N), as its contents in the remediated soil was brought down from 73.44 to 40.36 mg kg^−1^ dry mass, which accounts for 45.04%. In the case of PAH 3-cycle anthracene (A), during inoculation carried out in two collective series, the degree of reduction was within the range of 18.4–19.4%. Slightly greater reduction, in the range of 19.5–24.2%, was observed for PAH 4-cycle (BaA, CH). In the case of PAH containing five cycles in the molecule (BbF, BkF, BaP and DaA), the degree of reduction was already lower, within 12.0–20.7%. The reduction degree of 6-cycle PAH was the lowest among all PAHs (10.4–11.3%) (Figure 2). Providing the right amount of nutrients N and P for biostimulation and inoculation of appropriate degradators of petroleum hydrocarbons for bioaugmentation clearly influenced the number of appropriate degradators in soil contaminated with petroleum hydrocarbons, leading to a more effective degradation of TPH [9,13,22].

The use of biopreparation-1 during inoculation increased PAH reduction by 40.3%. It can be argued that the bacteria contained in biopreparation-1 are capable of biodegradation of TPH and PAH (*Bacillus subtilis*, *Gordonia terre*, *Rhodococcus erythropolis*, *Mycobacterium fredrikbergense* and *Pseudomonas fluorescens*) [17,18,22,67,68,69].

In order to increase the speed of biodegradation in the third series of inoculation, carried out for 50 days, the biopreparation-2 was used (biopreparation-1 modified by enrichment in non-pathogenic indigenous species of fungi and yeast) (Supplemental Table S2).

The results of the conducted analyses showed that the level of oil pollutants (Soil B4) was reduced to 1932 mg kg^−1^ dry mass. The highest reduction of aliphatic hydrocarbons was noted for n-alkanes with carbon length n-C_10_–n-C_23_ at levels of 61.3–85.1%. A lower degree of biodegradation was found for hydrocarbons with chain length n-C_23_–n-C_36_, whose contents in the soil were reduced to a satisfactory degree (by 27.5–64.0%) (Figure 1).

Evaluation indicators of biodegradation degree prove a satisfactory course of biological decomposition of n-alkanes, as they were significantly reduced in further stages of biodegradation: nC_17_/Pr from 15.075 to 0.097 and nC_18_/F from 8.771 to 0.126 (Table S3).

After modifying the composition of biopreparation-1 through its enrichment in non-pathogenic species of fungi (biopreparation-2), the third series of inoculation brought a higher degree of PAH reduction (from 75.68 to 26.80 mg kg^−1^ dry mass). As in the earlier stages of soil remediation, the highest degree of biodegradation was reached in the case of naphthalene (32.8%). A slightly lower degree of biodegradation was found for 3-cycle anthracene (A), at the level of 28.0%, while the level of content reduction of 4-cycle PAH (CH and BaA) was found within the limits of 28.1–31.3%. 5-cycle PAH (BbF, BkF, BaP and DaA) were reduced after inoculation (the 3rd series) by 17.5–23.1%. The degree of 6-cycle PAH content was the lowest of all PAHs, as it was within the limits 10.5–18.5% (Figure 2).

From the investigation of Soil B decontamination by ex-situ method, was found that the biopreparations used (particularly the biopreparation-2) were characterized by a relatively wide spectrum of PAH biodegradation, even of hydrocarbons with a higher number of aromatic rings in the molecule, which belong to the most carcinogenic and hardly biodegradable aromatic compounds (Figure 2).

Similar results were achieved by other researchers, who stated that in the initial stage of bioremediation, the bacterial community dominated the decomposition of saturated and partially aromatic hydrocarbons, and after days it turned out that the communities of fungi are dynamic and responsible for the degradation of polar hydrocarbons that create resistant metabolites [70].

First-order constants (k) in successive stages of the biodegradation process of petroleum pollutants grow: TPH (from 0.0089 to 0.0196 d^−1^), Σ nC_8_–nC_22_ (from 0.0099 to 0.0251 d^−1^) and Σ nC_23_–nC_36_ (from 0.0032 to 0.0122 d^−1^), PAH (from 0.048 to 0.141 d^−1^) (Table S4). They make it possible to trace and compare the kinetics of the biodegradation course of individual groups of petroleum pollutants (TPH, ∑n-C_8_–n-C_22_, ∑n-C_23_–n-C_36_, PAH) in the subsequent stages of soil treatment. Moreover, on the basis of the presented biodegradation constants, it is possible to compare the effectiveness of biopreparations based on indigenous bacteria and enriched with selected species of fungi and yeasts. The presented values of first-order biodegradation of petroleum pollutants in Soil B were similar to the results presented by many foreign researchers, who used similar models to describe the biodegradation of hydrocarbon pollutants [9,71,72].

After the process of biodegradation, Soil B4 was characterised by an elevated content of heavy metals (Zn, Pb, Cd) as well as naphthalene, chrysene and benzo(a)anthracene benzo(ghi)perylene, which are polycyclic aromatic hydrocarbons and constituted the research material in the phytoremediation processes.

#### 3.2.2. Ecotoxicological Assessment

During the biodegradation of petroleum hydrocarbons, chemical and microbiological changes may result in the generation of metabolites of differentiated or weakly recognized biological activity. For that reason, it is recommended to conduct toxicological testing which enables the monitoring of toxicity of the process of biodegradation of TPH and PAH in further stages of biodegradation of oil pollutants in Soil B in semi-technical conditions by the ex-situ prism method. The toxicity research was carried out by means of a set of new generation tests, whose bioindicators belong to various trophic levels.

**Figure 3:**
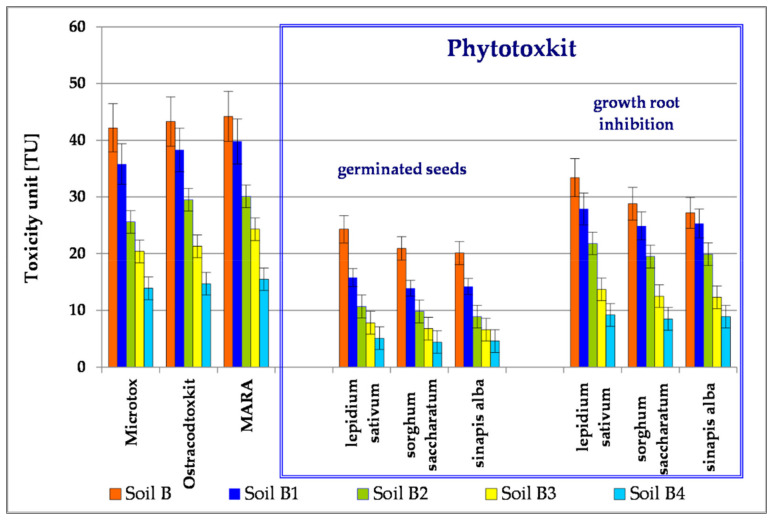
Comparison of the results of toxicity tests (expressed in TU toxicity units) of soil during the successive stages of biodegradation of petroleum pollutants—ex-situ method (repetition number *n* = 3, *p* < 0.05). (Soil B) raw soil; (Soil B1) soil after basic bioremediation; (Soil B2) soil after inoculation with I biopreparation (1st series); (Soil B3) soil after inoculation with I biopreparation (2nd series); (Soil B4) soil after inoculation with II biopreparation (3rd series).

The toxicological testing of reducers at the trophic level was conducted by means of the Microtox^®^SPT test, with application of the luminescent bacteria *Vibrio fischeri*. EC50, which causes 50% inhibition of luminescence of test bacteria in Soil B, amounted to a concentration of 2.37% of the total volume, which means the toxicity expressed by toxicity units is at the level of TU = 42.2. Toxicological tests carried out during successive stages of biodegradation of Soil B showed a drop in the degree of its toxicity: Soil B1 (TU = 35.8), Soil B2 (TU = 25.6), Soil B3 (TU = 20.4) and Soil B4 (TU = 13.4) (Figure 3).

The Ostracodtoxkit F^TM^ test, which was used in order to assess the toxicity of Soil B at the consumers’ level, is based on use of crustaceans *Heterocypris incongruens*, which are more sensitive to pollution than bacteria *Vibrio fisheri*. The results of the tests are presented in the Figure 3. Toxicity (TU) of Soil B amounted to 40.8 and was gradually decreased to TU = 12.5 after successive bioremediation processes.

Moreover, the innovative test MARA was carried out, which estimates environmental risk by using 11 test grafts of different sensitivity. The toxicity was calculated on the basis of an average toxic concentration MCT average and expressed in units of toxicity amounting to 38.2. During the biodegradation process of TPH and PAH, their level was brought down to 11.7 (Figure 3). The sensitivity of toxicological tests used is comparable, ranked in the order MARA < Ostracodtoxkit < Microtox.

In testing toxicity at the level of manufacturers, the phytotoxicity test Phytotoxkit^TM^ was used, in which the following plants are tested: *Lepidium sativum, Sorghum saccharatum* and *Sinapis alba.* After converting toxic concentrations into toxicity units (TU), taking into account plant germination as the criterion for the evaluation of toxicity, it was found that, during the conducted process of biodegradation of pollutants present in the tested soil samples, it was gradually reduced from 15.8 to 5.1 (*L. sativum*), from 13.9 to 4.4 (*S. saccharatum*) and from 14.2 to 4.6 (*S. alba*). For the second tested parameter—inhibition of root growth—the calculated values of TU for tested plants in Soil B were 25.3–27.9, after successive biodegradation stages (Soil B4) reduced to 8.5–9.2. The conducted tests showed that *L. sativum* (Figure 3) is the plant most sensitive to pollution in the test of soil samples.

### 3.3. Phytoremediation Soil

**Figure 4:**
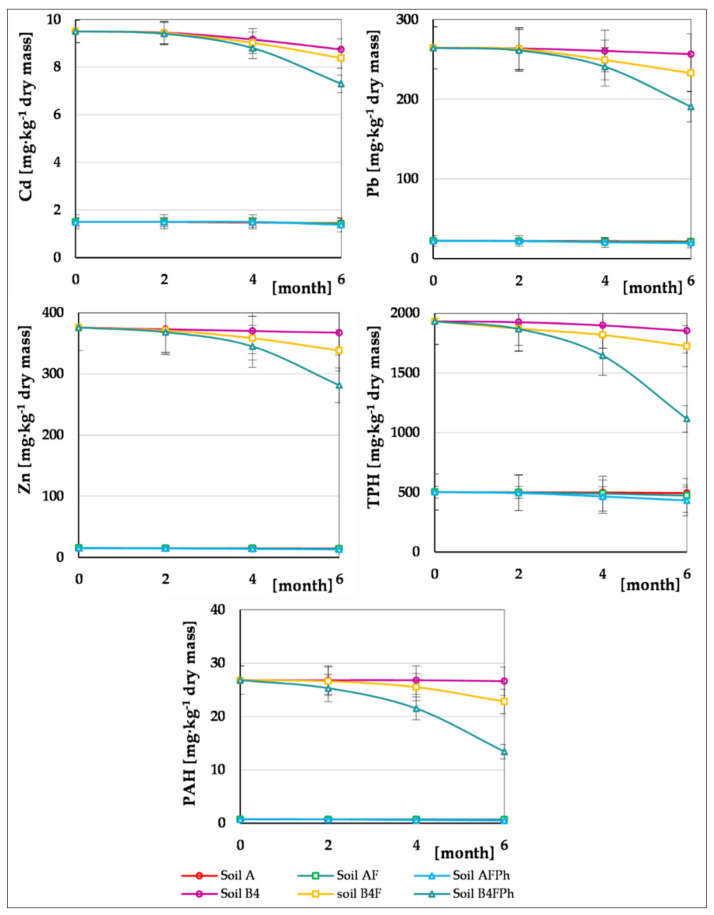
Comparison of reduction in the concentrations of Zn, Pb, Cd, petroleum hydrocarbons (TPH) and polycyclic aromatic hydrocarbons (PAH) during phytoremediation of soils: raw (A, B4), after fertilization (AF, B4F) and after phytoremediation carried out with *M. officinalis* (AFPh, BFPh) (repetition number *n* = 5–6, *p* < 0.05).

The results of this 6-month phytoremediation proved that soils contaminated with heavy metals show a very slow process of self-purification. Their rate of purification is to a considerable degree accelerated after phytoremediation with the use of the plant *Melilotus officinalis*, supported with the fertilization process, which was proved by monitoring Zn, Pb and Cd content in investigated soils during the experiment. The results of the examination are illustrated in Figure 4. On the basis of the obtained results, it can be stated that plant vegetation in Soil A, with insignificant amount of heavy metals (Pb, Ni, Cd), has an insignificant impact on their reduction. A slightly greater reduction was obtained by the addition of biogenic substances to Soil AF.

In Soil B4, after conducting biodegradable treatment of oil pollutants containing elevated levels of Zn, Pb and Cd, the 6-month phytoremediation process (assisted with the fertilizing process) obtained the following reductions: Pb from 264.4 to 190.7 mg∙kg^−1^ dry mass (27.9%), Zn from 375.8 to 281.4 mg∙kg^−1^ dry mass (25.1%) and Cd from 9.5 to 7.3 mg∙kg^−1^ dry mass (23.2%) (Soil B4FPh). The highest rate of heavy metal reduction in the soil subject to phytoremediation process was noted between 4th and 6th month (Figure 4).

**Figure 5:**
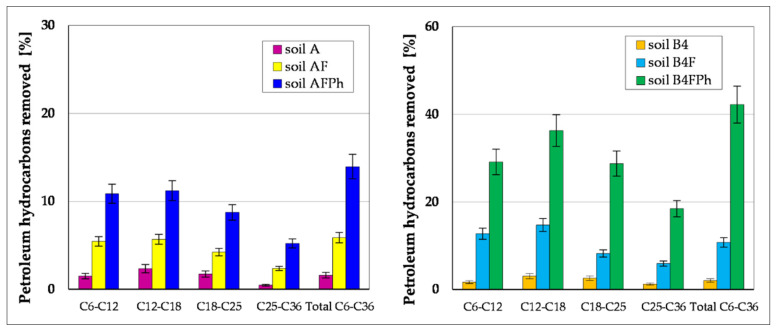
Comparison of the reduction in the content of specified groups hydrocarbons of TPH during phytoremediation of soils: raw (A, B4), after fertilization (AF, B4F) and after phytoremediation carried out with *M. officinalis* (AFPh, B4FPh) (*n* = 5–6, *p* < 0.05).

**Figure 6:**
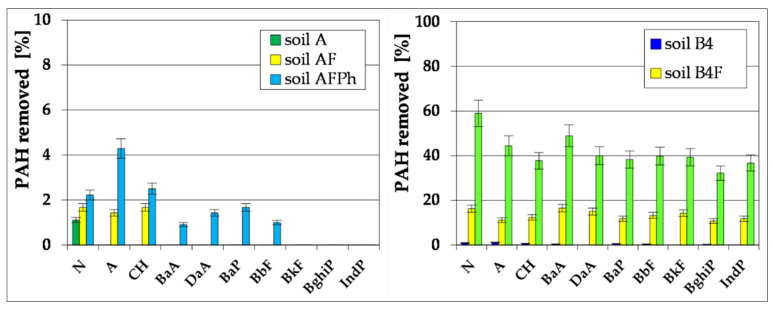
Comparison of the reduction in the content of individual PAH during phytoremediation of soils: raw (A, B4), after fertilization (AF, B4F) and after phytoremediation carried out with *M. officinalis* (AFPh, B4FPh) (repetition number *n* = 5–6, *p* < 0.05). Naphthalene (N), Anthracene (A), Chryzene (CH), Benzo(a)anthracene (BaA), Dibenzo(a,h)anthracene (DaA), Benzo(a)pyrene (BaP), Benzo(b)fluoranthene (BbF), Benzo(k)fluoranthene (BkF), Benzo(ghi)perylene (BghiP), Indeno(1,2,3-cd)pyrene (IndP).

During these tests, the possibility of using *M. officinalis* for phytoremediation to reduce oil pollutants which contaminate the soil was also analysed. Figure 5 and Figure 6 present the results of chromatographic analysis of petroleum hydrocarbons TPH and PAH in the soil samples during the 6-month experiment. Hydrocarbons of the carbon chain length from C_6_ to C_36_ were present in all samples. After termination of the experiment, the amount of hydrocarbons in each soil sample was reduced. Removal of individual groups of hydrocarbons proceeded in a descending order: (C_12_–C_18_ > C_6_–C_12_ > C_1_–C_25_ > C_25_–C_36_). In soils A and B4 their content was reduced by 1.6–4.1%, which may be caused by volatilization, elution, and photolysis of oil hydrocarbons [73]. A considerably higher reduction of petroleum hydrocarbons (TPH) was noted in the soil samples after the process of fertilizing, carried out by addition of the optimal amount of biogenic substances and correction of the pH reaction. These treatments contributed to activation of indigenous microorganisms, which support TPH reduction. After the 6-month period, their content was reduced in the soils, respectively, soil AF from 502.3 to 472.8 mg∙kg^−1^ of dry matter and soil B4F from 1932 to 1724 mg∙kg^−1^ dry mass. The highest reduction was observed for the group of hydrocarbons with carbon chain length C_12_–C_18_, which reached 5.7%, in Soil AF, whereas in Soil B4F, of higher TPH content, it was 14.7%. The lowest reduction content was observed for heavy hydrocarbons with carbon chain length C_25_–C_36_ within the range of 2.4–5.9%.

Conducting a 6-month process of phytoremediation with the use of *M. officinalis* supported by fertilization of tested soils shows that the TPH content was reduced in a much greater degree: in soil AFPh from 502.3 to 432.2 mg∙kg^−1^ dry mass (14.0%) and in soil B4FPh from 1932 to 1116 mg∙kg^−1^ dry mass (42.2%). The highest reduction of oil hydrocarbons was observed for the fraction C_12_–C_18_ (11.2–33.3%) and slightly lower for: C_6_–C_12_ (10.9–29.1%) and C_18_–C_25_ (8.8–28.8%). The content of other hydrocarbons from the range C_25_–C_36_ was reduced to a lesser degree, from 5.2–18.5% (Figure 5).

The data presented in Figure 6 depict the process of reduction of content of PAH, i.e., N, A, CH, BaA, DaA, BaP, BbF, BkF, BghiP and IndP in the tested soils. During the 6-month experiment, for each of the soils PAH content was reduced to different degrees. In Soils A and BF4, the total content of PAHs was reduced to an insignificant degree, whereas in the soils which were supplemented with biogenic compounds (AF and B4F) it was reduced within the range of 9.6–14.6%. In the result of the conducted 6-month phytoremediation process with the use of *M. officinalis* supported by fertilization, the PAH content was reduced by 31.5–49.9% (Soil BF4Ph). The highest degree of hydrocarbons reduction was reached for naphthalene, anthracene and chrysene (37.5–51.1%), whereas reduction of the remaining PAHs ranged from 24.0 to 30.6%.

#### Plant Material Analysis

**Figure 7:**
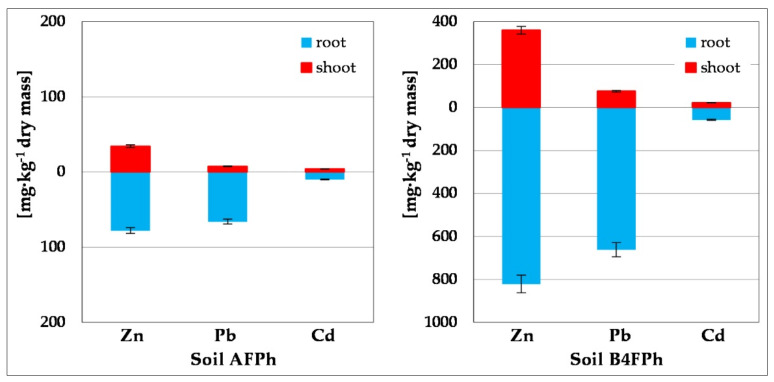
Concentration of Zn, Pb and Cd in the biomass (shoots and roots) after 6-month phytoremediation enhanced by fertilization of soils AFPh and B4FPh (repetition number *n* = 3, *p* < 0.05).

After termination of the 6-month process of phytoremediation of contaminated soils conducted with the use of *M. officinalis*, the plant material was collected and, after cleaning from soil and separation of the shoots from roots, it was dried. The resulting dry matter of roots and shoots were mineralized, and levels of metals Zn, Pb and Cd were measured. The results of these tests are illustrated in Figure 7, whereas the calculated values of coefficients characterizing their ability to take up heavy metals from soil (Zn, Pb, Cd) are presented in Table 2. Concentrations of heavy metals in roots and shoots for *M. officinalis* growing in Soil 4BFPh were determined in descending order: Zn > Cd > Pb. Metal content was much higher in roots than in shoots of all cultivated plants. Metal concentrations in roots were 822, 662 and 57 mg∙kg^−1^ dry mass, whereas in shoots 360, 76 and 23 mg∙kg^−1^ dry mass, respectively, for Zn, Pb and Cd. As is shown by the values of the coefficient TF (Table 2), zinc was the most translocated element (TF = 0.44), whereas lead and cadmium were less translocated from roots to shoots: Pb (TF = 0.12) and Cd (TF = 0.40). The coefficients of metal bioconcentration show dependence BCF_roots_ > BCF_shoots_ and amount, respectively, to Zn (2.19 > 0.96), Pb (2.50 > 0.29), Cd (6.02 > 2.42), what shows that the plant *M. officinalis* accumulates metals mostly in roots, behaving thus as phytostabiliser. In the case of using of *M. officinalis* in phytoremediation of soils AFPh (of low metal content), a similar trend in ability to take up metals from soil was noted in the order Zn > Pb > Cd. Lead was the least likely to be transferred from roots to shoots.

These tests show that higher contamination of soil with heavy metals causes a significant increase of their content both in roots and shoots of plants. The analysis of the plant material *M. officinalis* showed a higher degree of metal accumulation in root parts. Moreover, one can notice higher accumulation of zinc in comparison with the remaining metals.

### 3.4. Toxicological Tests of Soil

#### 3.4.1. Phytotoxkit^TM^

**Figure 8:**
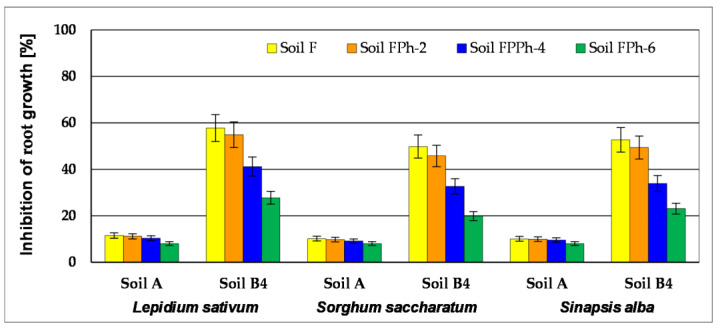
Comparison of root growth inhibition (*Lepidium sativum*, *Sinapis alba*, *Sorghum saccharatum*) in the Phytotoxkit^TM^ test during soil phytoremediation (repetitions number *n* = 3, *p* < 0.05).

In testing toxicity at the level of manufacturers, the standard Phytotoxkit^TM^ test was used, which is a test of chronic toxicity based on assessment of germination and early growth of plants. The following plants were tested: *Lepidium sativum, Sorghum saccharatum* and *Sinapis alba*. Phytotoxicity tests were carried out on soil samples after their fertilisation (AF and B4F) and in soils after the process of phytoremediation supported with fertilisation with the use of *M. officinalis* (AF Ph and B4F Ph). The phytotoxicity tests were conducted at the beginning of the experiment and after 2, 4 and 6 months (termination of phytoremediation). The results of these tests were compared to the results obtained from growth of tested seeds in the control soil (delivered by the producer of the Phytotoxkit^TM^ test). The results of the tests are specified in Table 3 and illustrated in Figure 8. The test conducted for the soil with low concentration of heavy metals (Zn, Pb, Cd) in soil AF showed that the amount of germinated seeds in the initial stage of the experiment was for *L. Sativum* 93% and both *S. alba* and *S. saccharatum* 97%. After 6 months of phytoremediation, the results of the conducted tests showed that the seeds of all tested plants germinated at a rate of >99%, whereas inhibition in root growth was reduced for *L. sativum* (from 11.5 to 10.4%), *S. alba* (from 10.1 to 9.6%) and *S. saccharatum* (from 10.2 to 9.2%).

The test of phytotoxicity for Soil B4B—containing higher quantities of Zn, Pb, Cd and hydrocarbon pollution left after biodegradation processes of Soil B (basic bioremediation and inoculation with biopreparations on the basis of non-pathogenic species of bacteria, fungi and yeast) TPH (1931.9 mg∙kg^−1^ dry mass) and PAHs (26.8 mg∙kg^−1^ dry mass)—showed that the amount of germinated seeds is considerably lower in comparison to Soil A, amounting to 55% in *L. sativum*, 67% in *S. alba* and 60% in *S. saccharatum*. Inhibition of root growth was at the following levels: *L. sativum*—58.7%, *S. alba*—49.8% and *S. saccharatum*—52.7%.

After the test of phytoremediation, a reduction of toxic properties in the contaminated soil was noted, as within the period between 2 and 6 months of the phytoremediation process there was an increase in the number of germinated seeds (*L. sativum* from 68 to 90%, *S. saccharatum* from 79 to 97% and *S. alba* from 75 to 93%) and a decrease in inhibition of the root growth (*L. sativum* from 54.9 to 27.8%, *S. saccharatum* from 45.8 to 19.7% and *S. alba* from 49.4 to 23.1%) (Table 3, Figure 8).

#### 3.4.2. Ostracodtoxikit F^TM^

Monitoring of toxicity of non-contaminated soil A and contaminated soil B4 during phytoremediation, which was carried out with the Ostracodtoxikit F^TM^ test, showed that Soil A was not toxic for crustaceans *Heterocypris incongruens*, which serve as bioindicators in the test. Mortality of crustaceans during the remediation process of Soil A was within the limits of 6.7% in the initial stage of the process (Soil AF) to 3.3% after its termination (Soil AF Ph-6), whereas inhibition of growth was noted within the limits of 5.8 to 4.1%.

**Figure 9:**
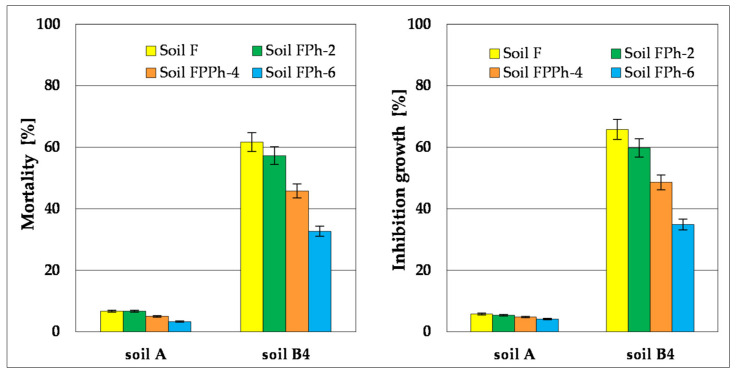
Comparison of death rate and growth inhibition (*Heterocypris incongruens*) during phytoremediation carried out with *Melilotus officinalis*, enhanced by fertilization of soils A and B4 (repetitions number = 3, *p* < 0.05).

Investigation of toxicity of contaminated soil (Soil B4) showed its considerable toxicity for *Heterocypris incongruens* in the 6-days test. Before starting the process of phytoremediation (Soil B4F) in contact with crustaceans caused their mortality to reach 61.7%, while growth was inhibited by 65.8%. The process of phytoremediation of soil caused a decrease in its toxicity, whose dynamic grew proportionally to the phytoremediation factor—*M. officinalis*. After two months of phytoremediation, Soil B4 F Ph-2 in contact with bioindicators caused a mortality rate of 57.3% and an inhibition in growth of 59.8% The six-month process of phytoremediation caused a significant decrease in toxicity of Soil B4 FPh-6, as during its contact with tested crustaceans a decrease in mortality from 61.7 to 32.7% was observed, along with a decrease in growth inhibition of *Heterocypris incongruens* from 65.8 to 34.9% (Figure 9). Reduction of toxic impact of soils on crustaceans *Heterocypris incongruens* confirms progress with time of phytoremediation and reduction of contamination.

#### 3.4.3. Microtox^®^SPT

Assessment of the degree of contamination of soils during the process of phytoremediation with the use of *M. officinalis* as a phytoremediation agent was accomplished by use of the Microtox^®^SPT test. The results of reduction of luminescence of the bacteria *Vibrio fischeri*, tested in contact with soils on the basis of which the coefficient EC50 was calculated, are specified in the Table 4. In the initial phase of the experiment and after 2, 4 and 6 months, it was shown that Soil AF was not toxic for bacteria *V. fischeri*. Soil B4, contaminated by (TPH and PAH) containing elevated contents of Zn, Pb and Cd in the initial stage of phytoremediation and showing inhibition of the luminescence of bacteria *V. fischeri* corresponding to the level TU = 13.5, the phytoremediation process caused reduction in the soil toxicity. After two months of the experiment, the soil (B4Ph-2) showed toxicity at the level of TU = 12.2 and after 4 months (Soil BFPh-4) TU 8.5, whereas toxicity of the soil after termination of phytoremediation (Soil BF Ph-6) was reduced to TU = 5.8 (Table 4).

#### 3.4.4. The MARA Test

**Figure 10:**
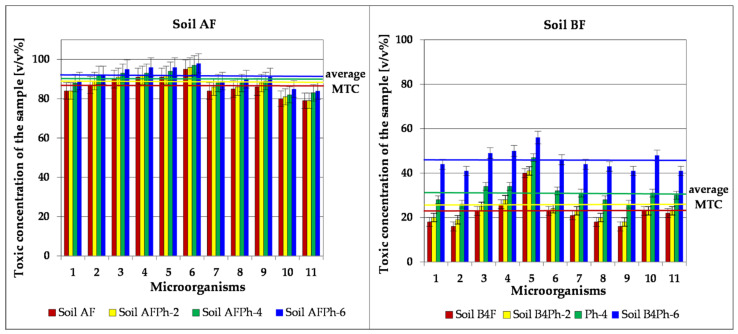
Environmental risk assessment test MARA for soils A and B4 during 6 months of phytoremediation augmented by soils fertilisation AF and B4F with microorganisms (1. *Microbacterium spaciec*, 2. *Brevundimonas diminuta*, 3. *Citrobacter freudii*, 4. *Comamonas testosteroni*, 5. *Entrococcus casseliflavus*, 6. *Delftia acidovorans*, 7. *Kurthia gibsoni*, 8. *Staphylococcus warneri*, 9. *Pseudomonas aurantiaca*, 10. *Serriatia rudidaea*, 11. *Pichia anomala*), (MTC) Microbial Toxic Concentration (repetitions number *n* = 3, *p* < 0.05).

The MARA (Microbial Assay for Risk Assessment) test is an innovative assessment of environmental risk, in which the system of assessment of chronic toxicity of samples uses as bioindicators ten prokaryotic organisms (bacteria of various taxonomy) and one eukaryotic (yeast). Figure 10 presents values of MTC Microbial Toxic Concentration assayed in the MARA test—concentrations toxic for tested microorganisms due to their contact with soil extracts. The investigation was made on soil samples before the process of phytoremediation (AF and B4F), after 2 months (AFPh-2 B4FPh-2), 4 months (AFPh-4 and B4F Ph-4) and 6 months (AFPh-6 and B4FPh-6).

For a non-contaminated soil sample (Soil AF), the mean value of toxic microbiological concentration (MTC average) was of 86.5% *v*/*v*, whereas the lowest toxic concentration was MTC_min._ = 79% *v/v* for strain no.11. A high resistance (MTC: 90–95% *v*/*v*) was shown by grafts no. 3, 4, 5 and 6. The results of the MARA test show that phytoremediation of soil slightly reduced its toxicity in successive months of the experiment. After two months, (AFPh-2) MTC average assayed for the soil is 87.6% *v*/*v*, after four months (AF Ph-4) MTC average = 89.7% *v*/*v* and after six months (AF Ph-6) MTC average = 91.4% *v*/*v*. For the soil after phytoremediation, the lowest toxic concentration was 84% *v*/*v* and the graft no. 6, for which MTC = 98% *v*/*v* proved to be the most resistant organism.

For the Soil B4F with elevated content of Zn, Pb, Cd, TPHs and PAHs, the medium toxic concentration was 22.2% *v*/*v*, whereas the lowest toxic concentration was found in grafts no. 2 and 9 (MTC_min._ = 16% *v*/*v*) and the highest for microorganism no. 5 (MTC_max._ = 40% *v*/*v*). The MARA test conducted every two months for the soil decontaminated by means of phytoremediation showed successive decreases of its toxicity. After 2 months, the average toxic concentration in the purification soil (BF Ph-2) was 24.2% *v*/*v*, after 4 months for Soil BF Ph-4 the MTC average = 31.5% *v*/*v* and for the soil after termination of the experiment (BF Ph-6) the MTC average = 45.7% *v*/*v*. For this type of soil, it was proven that the highest resistance (toxic concentration above 45% *v*/*v*) was shown by grafts no. 3, 4, 5, 6 and 10, and the most sensitive were microorganisms no 2, 9 and 11 (MTC = 41% *v*/*v*) (Figure 10).

## 4. Discussion

Pollutants in the soil prompt interaction with the environment, change it chemically and physically, and contribute to biological imbalance. Resistance of soil to degradation caused by hydrocarbons, heavy metals and other substances depends upon many factors, such as physical and chemical properties of the soil, concentration and type of chemical pollutants, content of biogenic compounds (nitrogen, phosphorus), pH value of the soil, concentration of organic compounds and quantitative and qualitative composition of microorganisms present in soil [63,74,75]. Having optimal levels of biogenic components in soils has tremendous significance during phytoremediation of contaminated soils. Carrying out the fertilization treatment stimulates plant growth during phytoremediation of soils contaminated with heavy metal [63]. The speed of biodegradation of hydrocarbons is limited by the content of biogenic substances, and their introduction to soils being decontaminated accelerates the metabolism of indigenous microorganisms which take part in the biodegradation process [6,9].

Phytoremediation is a balanced technology able to effectively removal small or moderate contamination. However, complex contamination may drastically decrease efficiency, as the plants may prove to be sensitive to organic contamination, growing slowly and thus weakening the absorption of metals. In a case when the activity of indigenous bacteria which decompose hydrocarbons and foster growth of plants is not sufficient, more sophisticated strategies may be required [27,76].

High content of petroleum pollutants in the soil from weathered drill wastes G-6 creates a necessity to use their reduction in successive steps of purification technology, which comprises basic bioremediation and inoculation with biopreparations on the basis of non-pathogenic species of indigenous bacteria, which in the final stage of inoculation was enriched in selected non-pathogenic species of fungi and yeast for biodegradation of pollutants which are difficult to decompose biologically [1,2,6].

The task of the stage comprising basic bioremediation was to increase the number of indigenous microorganisms by enrichment of the environment in nutrients and optimisation of process parameters [7,8,9]. Soil B, which belongs to so-called “heavy” soils featuring lower demand for biogenic substances than “light” soil, was treated by determining mutual dependencies between the dehydrogenase activity of soil and the amount of added biogenic substances in line with decreases of petroleum pollution in the soil being purification. A similar method of selection of biogenic substances was also used in other investigations by other scientists [77,78]. Basic bioremediation in laboratory conditions (method ex-situ) resulted in a considerable reduction of petroleum pollution—TPH by 33.9%—along with a slight reduction of PAH by 9.5% (Soil B1). Implementation of inoculation with biopreparation-1 on the basis of non-pathogenic indigenous bacteria allowed for decrease of TPH content by 86.3% and PAH by 40.3%. Chromatographic analysis allowed for a wider view on the course of the process, as it enabled the determination of susceptibility to biodegradation of particular groups of hydrocarbons. The speed of removal of particular groups of hydrocarbons was, in descending order, nC_8_–nC_22_ > nC_23_–nC_28_ > nC_29_–nC_36_. This scheme of decontamination is probably linked to the chemical structure of alkanes. Alkanes of the chain length C_10_–C_22_ are the substances most likely used by bacteria in metabolic processes [6,8,9,11,12]. The relatively visible degree of PAH biodegradation decreases as the number of rings in a molecule increase. Reduction in PAH content proves that inoculation with biopreparation-1 (Table S2) contains bacteria which have biodegradation abilities for aromatic hydrocarbons, which was proven by laboratory tests and data from the literature [12,14,16,17,18,20,67,68,69,70,79]. Modification of the biopreparation-1 by enriching it in indigenous non-pathogenic species of fungi and yeast (*Trichoderma asperellum*, *Phanerochaete chrysosporium* and *Candida oleophia*) (biopreparation-2) and its use in the 3rd series of inoculation allowed for an increase in the degree of biodegradation of aliphatic hydrocarbons with long carbon chains and, in particular, PAH by further 28.9%, which indicates biodegradation abilities of selected species of fungi and yeast, confirmed by other researchers [23,70]. The calculated biodegradation constants of the 1st order allowed for investigation and comparison of kinetics of biodegradation course of particular groups of petroleum pollutants (TPH, Σn-C_8_–n-C_22_, Σ n-C_23_–n-C_36_, PAH) (Supplemental Table S4) in further stages of soil purification. The obtained results are consistent with the results of other researchers [71,72,80,81]. Such approach allows us to trace the whole putrification process, to determine effectiveness of biodegradation of particular groups of hydrocarbons in the use of various biopreparations, and to confirm usefulness of introduction of the biopreparations enriched in fungi and bacteria in the final stage of purification. Validity of the used concept of stage-wise purification of Soil B has been confirmed by toxicological tests of degree of toxicity expressed in units (TU), which was proven by these tests: Microtox^®^ (from 42.1 to 13.9), Ostracodtoxkit F^TM^ (from 43.3 to 14.7) and MARA (from 44.2 to 15.5). The performed toxicological studies indicate that the sensitivity of the applied toxicological tests is comparable and increases in the following order: MARA < Ostracodtoxkit F^TM^ < Microtox^®^. Other researchers also noted a lower sensitivity of the MARA test [49]

The procedures used, along with the gradual initial treatment (basic bioremediation and inoculation with biopreparations on the basis of indigenous specious of bacteria, fungi and yeast), contributed to reduction of the level of petroleum pollution (TPH and PAH) and significantly improved soil fertility, which allows the plants to extract metals. Moreover, it increases populations of microorganisms oxidizing hydrocarbons in the soil, which may be activated and used in successive stages of purification.

It was assumed that the next stage of purification of Soil B4, containing heavy metals (Zn, Cd and Pb) and remaining petroleum pollutants (TPH and PAH), would be the process of phytoremediation. In this method, the key factor is to select a proper plant, which shows tolerance during vegetation to contamination in the soil. From the plants which cause reduction in quantity of heavy metals and petroleum pollutants in soil during their growth, *M. officinalis* was selected. This plant has been successfully used in phytoremediation processes when cleaning soil from heavy metals and organic substances from soil [26,27,36,43,44,82]. The tests showed that, during phytoremediation of soils, the effect of their purification depended upon the type and concentration of pollutants (heavy metals, TPH and PAH), and it proceeded at different rates depending upon the type and dose of toxicant.

In order to assess the abilities of *M. officinalis* to accumulate metals taken up from soil in tissues and transfer them from roots to shoots, coefficients TF and BCF have been calculated. The values of translocation coefficients TF were for Zn 0.44, Pb 0.12 and Cd 0.40. The calculated coefficients of concentration BCF (BCF_roots_ > BCF_shoots_) prove that heavy metals taken up by *M. officinalis* are accumulated mainly in root tissues in the following order: Zn > Pb > Cd, revealing a weak translocation of metal from roots to shoots. The high accumulation of trace elements in plant tissues suggests a possibility of their negative impact on the activity of microorganisms, and thus it may distort functioning of the ecosystems of contaminated areas [82].

Chromatographic analyses conducted to determine TPH and particular groups of hydrocarbons in investigated soils, carried out before and after their phytoremediation, showed significant pollution decreases. Phytoremediation of soil with low content of TPH (Soil AF) caused reduction of this group of compounds by 14.0%, whereas for the soil with initial TPH content—1932 mg kg^−1^—(Soil B4F) phytoremediation process reduced its content in soil to 1116.0 mg kg^−1^ dry mass, which accounts for 42.2%. The speed of removal of particular groups of hydrocarbons was in the sequence C_12_–C_18_ > C_6_–C_12_ > C_18_–C_25_ > C_25_–C_36_. This scheme of removal is probably connected with chemical structure of alkanes. Hydrocarbons of shorter length carbon chain are more prone to degradation, whereas hydrocarbon fractions with longer carbon chains show higher hydrophobicity and separator factor octanol-water (Kow) and are less bio-accessible because of higher sorption of organic matter [83]. Alkanes of chain length C_10_–C_22_ are the substances most preferred by bacteria in metabolic processes. Moreover, it may be expected that, apart from hydrocarbon chain length, susceptibility to degradation is influenced by chain branching, presence and placing of substituents in the molecule, structure and number of rings [11,12,84].

The conducted experiment of phytoremediation of soils with the use of *M. officinalis* showed high speed of reduction in TPH after the fertilization process (Soil AF—5.9%, Soil B4F—0.8%). This likely takes place as a result of stimulation of microorganisms in the rhizosphere. These experiments confirm the active role played by *M. officinalis* in the rhizosphere as a result of the operation of plant enzymes released in root exudates, rich in such organic compounds as amino acids, organic acids, sugars, enzymes and complex carbohydrates which provide source of carbon and energy for growth of rhizosphere microorganisms, which foster degradation of hydrocarbons [27,36,41,43,85,86]. In addition, not insignificant is soil loosening connected with penetration of roots during their growth, which improves bioavailability and increases the possibility of using the biodegradation potential of microorganisms in soil purification [63,87].

In the case of removal of polycyclic aromatic hydrocarbons from soil, efficiency of *M. officinalis* for the process of phytoremediation was proven. In soil with trace amounts of PAH (Soil AF), the process of phytoremediation caused insignificant reduction (from 0.73 to 0.66 mg kg^−1^ dry mass). A higher degree of PAH reduction (14.9%) was obtained during phytoremediation of Soil B4F, in which naphthalene, chrysene, benzo(a)anthracene and benzo(ghi)perylene dominated. As a result of the 6-month phytoremediation, their concentration in Soil B4FPh was decreased: naphthalene from 13.56 to 5.56 mg kg^−1^ dry mass, chrysene from 2.76 to 1.72 mg kg^−1^ dry mass, benzo(a)anthracene from 4.56 to 2.33 mg kg^−1^ dry mass and benzo(ghi)perylene from 3.08 to 2.09 mg kg^−1^ dry mass. The findings given in the literature prove the efficiency of *M. officinalis* in phytoremediation processes of soils contaminated with PAH [24,25,27,46].

Toxicological monitoring conducted during phytoremediation with the use of *M. officinalis* showed that integrated chemical and toxicological analyses make possible a precise identification of investigated soils. In the toxicological monitoring of phytoremediation process of soils, a package of toxicological tests was used, in which bioindicators belonged to three taxonomic groups: producers (Phytotoxkit^TM^), consumers (Ostracodtoxkit F^TM^) and reducers (Microtox^®^STP and MARA), which allowed us to assess the state of the investigated soil environment. Biotest packages for conducting environmental analyses and assessment of changes in toxicity in remediation processes were used also by other researchers [48,49,51,52].

Toxicological monitoring of phytoremediated tests was conducted at the beginning of the experiment and after two, four and six months of phytoremediation of soils, revealing continuing decreases of toxicity proportional to the biomass of the phytoremediating agent, which was *M. officinalis*.

The Phytotoxkit^TM^ test of phytotoxicity showed that *L. sativum* had the highest sensitivity to contamination, whereas *S. alba* and *S. saccharatum* had slightly lower sensitivity to toxicants. For less-polluted soils AF, after phytoremediation with the use of *M. officinalis*, the amount of germinated tested plants was 100%, whereas inhibition of root growth decreased from 10.1–11.5% to 9.2–10.4%. In the case of soils BF, contaminated with heavy metals, TPHs and PAHs, the phytoremediation process caused an increase in the amount of germinated seeds in Soil BFPh-6 in the ranges of 54.9–66.9% to 90.4–96.7% and decreases in the inhibition of root growth from 49.8–58.7% to 19.7–27.8%. Phytotoxkit^TM^ is a test used in toxicological monitoring by many researchers [51,52,53,63].

The results of the Ostracodtoxkit F^TM^ microbiotest, carried out for soils subject to decontamination, confirmed the efficiency of the phytoremediation process. Moreover, this test showed that the soil taken from the forested area in the vicinity of weathered drill wastes (Soil AF) is not toxic for crustaceans *H. incongruens*, which are bioindicators in this test, whereas the test conducted for soils B4F showed their significant toxicity in the initial phase of the test, which decreased during the process of phytoremediation. The obtained reduction in mortality of *H. incongruens* from 61.7% (Soil B4FPh) to 32.7% (Soil B4FPh-6) and inhibition of their growth from 65.8 to 34.9% demonstrates that, with time, the phytoremediation process leads to a reduction in contamination.

Similar results were obtained after conducting the Microtox^®^SPT test, the results of which, conducted for the soil AF, showed its insignificant toxicity (TU = 2.5). After 6 months of phytoremediation, this soil, did not show toxicity for the bio-indicators *V. fischeri*. The Microtox^®^SPT test for Soil B4F, contaminated with Zn, Pb and Cd as well as TPHs and PAHs, showed that it is significantly more toxic (TU = 13.5). The process of phytoremediation reduced its toxicity to TU = 5.8.

The satisfactory effect of phytoremediation of soils with the use of *M. officinalis* is confirmed by the results of the MARA test, which contains as indicators various bacteria and yeast [55,56,65]. This test showed insignificant influence of Soil AF on tested microorganisms, which proves that it is not toxic, whereas for the contaminated soils B4F we observed a low mean value of microbiological concentration (MTC average = 22.2 *v*/*v*%), entailing elevated toxicity. The MARA test conducted for investigated soils during phytoremediation proved the efficacy of the experiment, as after its termination the MTC of Soil B4FPh-6 was 45.7 *v*/*v*%.

All conducted toxicological tests showed reduction of toxicity of soils phytoremediated with the use of *M. officinalis* as a phytoremediating agent, which correlates with reduction of harmful pollutants contained in soils which were assayed by analytical methods.

The above mentioned tests confirmed the efficacy of the technologies, in which advanced phytoremediation was used in combination with other biological approaches to reclaim soils containing high concentrations of oil pollutants occurring in combination with heavy metal pollution. Moreover, the presented results of the investigation demonstrate the advisability of extending soil testing after particular stages of purification for ecotoxicity and phytotoxicity. Accumulation of analytical and toxicological data relating to quality of soils not only has an important cognitive aspect, but also practical use. It allows for a comprehensive assessment of soils, ensuring their safer introduction into the environment.

## 5. Conclusions

The aim of the study was to assess the possibility of using the *Melilotus officinalis* plant in the phytoremediation of soils contaminated with heavy metals (Zn, Pb, Cd) and coexisting petroleum hydrocarbons (TPH and PAH). These pollutants remained after the conducted soil bioremediation processes.

It has been shown that the use of the technology involving basic bioremediation and inoculation with biopreparations based on autochthonous non-pathogenic species of bacteria, fungi and yeasts allowed for a gradual reduction of petroleum hydrocarbons (TPH and PAH) in the soil to the level enabling the phytoremediation using the plant *M. officinalis*.

As a result of the soil treatment process, a reduction in the content of pollutants was achieved: TPH from 56,371 to 1932 mg kg^−1^ dry mass, PAH from 139.28 to 26.80 mg kg^−1^ dry mass, Zn from 395.2 to 375.8 mg kg^−1^ dry mass, Pb from 285.6 to 264.4 mg kg^−1^ dry mass, Cd from 11.0 to 9.5 mg kg^−1^ dry mass. Soil phytoremediation has reduced the pollutant content: TPH from 1932 to 1116 mg kg^−1^ dry mass, PAH from 26.8 to 13.4 mg kg^−1^ dry mass, Zn from 375.8 to 281.4 mg kg^−1^ dry mass, Pb from 264.4 to 190.7 mg kg^−1^ dry mass, Cd from 9.5 to 7.3 mg kg^−1^ dry mass.

Tests of the plant material showed that heavy metals taken up by *Melilotus officinalis* were mainly accumulated in the root tissues (BCF_roots_ > BCF_shoots_) in the following order Zn > Pb > Cd, revealing a poor metal translocation from the roots to the shoots, which indicates that *M. officinalis* behaves as a phytostabilizer.

Extensive toxicological monitoring, which is an element of novelty, carried out during the process of biodegradation of petroleum pollutants (TPH and PAH) and phytoremediation with the use of *M. officinalis* allows to track changes in toxicity that correlate with the reduction of pollutants (Zn, Pb, Cd, TPH and PAH) in the tested soils.

The performed toxicological studies indicate that the sensitivity of the applied toxicological tests is comparable and increases in the following order: MARA < Ostracodtoxkit F < Microtox.

The obtained research results confirmed the correctness of the adopted concept of applying gradual soil treatment in biological processes (bioremediation and phytoremediation with the use of *Melilotus officinalis*) for the treatment of soil contaminated with heavy metals and coexisting petroleum hydrocarbons (TPH and PAH).

## Figures and Tables

**Table 1:** Parameters of the chromatographic method for the determination of TPH and PAH on the Clarus 500 GC gas chromatograph.

Parameter of the Chromatograph					Calibration Standards
Temperature (°C)			Capillary Coumn	Carrier Gas	
Injector	Detector	Temperature Program			
Chromatographic analysis of aliphatic hydrocarbons—TPH, nC_6_—nC_44_, pristane, phytane (Clarus 580 GC)					
290 °C	FID, 300 °C	28 °C—isothermal run in 2 min 28–105 °C—temp. increase rate 10 °C min^−1^ 105–285 °C—temp. increase rate 5 °C min^−1^ 285 °C—isothermal run in 10 min^−1^	Quadrex 007-1 30 m × 0.53 mm	He, 20 mL min^−1^	reference soil: BAM—K010 Supelco No. D2807 (nC_6_–nC_40_) Restek No. A029668 (Fuel Oil Degradation Mix nC_17_, pristane, nC_18_, phytane)
Chromatographic analysis of polycyclic aromatic hydrocarbons—PAH (Clarus 500 GC)					
320 °C	FID, 320 °C	40 °C—isothermal run in 2 min 40–240 °C—temp. increase rate 30 °C min^−1^ 240–320 °C—temp. increase rate 8 °C min^−1^ 320 °C—isothermal run in 10 min^−1^	RTX-440 30 m × 0.25 mm	H_2_, 8 mL min^−1^	Reference soil containing 16 PAHs: BAM—ERM-CC013 Mixture of 16 PAHs in chloroform: nr 31011 (2000 μg ml^−1^ each), nr 31264 (500–1000 μg ml^−1^), nr 31451 (100–200 μg ml^−1^) made by Restek

**Table 2:** Parameters describing the capacity, efficiency and usefulness of *M. officinalis* after 6 months of phytoremediation (Zn, Pb, Cd) augmented by soils fertilisation AFPh and B4FPh (repetitions number *n* = 3, *p* < 0.05).

Parameter	Soil AFPh			Soil B4FPh		
	Zn	Pb	Cd	Zn	Pb	Cd
TF	0.44 ± 0.02	0.12 ± 0.01	0.40 ± 0.02	0.44 ± 0.02	0.11 ± 0.01	0.40 ± 0.02
BCF_shoot_	2.30 ± 0.12	0.34 ± 0.02	2.62 ± 0.12	0.96 ± 0.08	0.29 ± 0.03	2.42 ± 0.18
BCF_root_	5.23 ± 0.32	2.96 ± 0.16	6.53 ± 0.45	2.19 ± 0.16	2.50 ± 0.18	6.02 ± 0.60

**Table 3:** Comparison of the amount of germinated seeds (*Lepidium sativum*, *Sinapis alba*, *Sorghum saccharatum*) in the Phytotoxkit^TM^ test during phytoremediation with *Melilotus officinalis* enhanced by fertilization of soils A and B4 (repetitions number *n* = 3, *p* < 0.05).

Germination (%)	Soil A				Soil B4			
	AF	AFPh-2	AFPh-4	AFPh-6	B4F	B4FPh-2	B4FPh-4	B4FPh-6
*Lepidium sativum*	93 ± 1	97 ± 1	97 ± 1	>99 ± 1	55 ± 3	68 ± 3	84 ± 2	90 ± 1
*Sorghum saccharatum*	97 ± 1	>99 ± 1	≥99 ± 1	>99 ± 1	67 ± 3	79 ± 2	90 ± 1	97 ± 1
*Sinapis alba*	97 ± 1	97 ± 1	>99 ± 1	>99 ± 1	60 ± 3	75 ± 2	90 ± 1	93 ± 1

**Table 4:** Comparison of EC50 and TU values during phytoremediation carried out with *M. officinalis*, enhanced by fertilization of soils A and B according to the Microtox^®^SPT test (repetitions number *n* = 5, *p* < 0.05).

Parameter	Soil A				Soil B4			
	AF	AFPh-2	AFPh-4	Ph-6	B4F	B4FPh-2	B4FPh-4	B4FPh-6
EC 50 [mg soil dm^−3^]	35,712 ± 3687	50,369 ± 5032	N	N	6451 ± 728	6971 ± 815	10,556 ± 1136	14,759 ± 1680
TU	2.5	1.9	–	–	13.5	12.2	8.5	5.8

N—no toxicity.

## Data Availability

All supporting data have been included in this study and are available from the corresponding authors upon request.

## Supplementary Materials

The following are available online at https://www.mdpi.com/article/10.3390/toxics9070148/s1, Figure S1. Schema of the purification of soil taken from G-6 waste pit (Soil B), led in semi-technical conditions (bioremediation—ex situ prism method, phytoremediation—pot tests), Figure S2. Scheme of the test stand for conducting the process of biodegradation of pollutants (TPH and PAH) in soil under semi-technical conditions (*ex situ* prism method), Table S1: Chemical and physical properties of the soil, Table S2: Species status of microorganisms strains included biopreparations, Table S3: Indicators of n-alkane biodegradation degree after consecutive purification stages of waste from soil B (ex-situ method), Table S4: Equation coefficients of mathematical model biodegradation of TPH and PAH in consecutive stages of soil B treatment (laboratory conditions, ex-situ method).
