# Supplementary material for: Assessment of the Suitability of Melilotus officinalis for Phytoremediation of Soil Contaminated with Petroleum Hydrocarbons (TPH and PAH), Zn, Pb and Cd Based on Toxicological Tests
Source: Toxics. 2021 Jun 25;9(7):148. doi: 10.3390/toxics9070148 (PMC8309879; doi:10.3390/toxics9070148)
Supplement: Supplementary file 1 [file toxics-09-00148-s001.zip › toxics-1252142-supplementary.pdf]

toxics

MDPI

Article

Assessment of the suitability of Melilotus officinalis for phytoremediation of soil contaminated with petroleum hydrocarbons (TPH and PAH), Zn, Pb and Cd based on toxicological tests

Teresa Steliga, Dorota Kluk

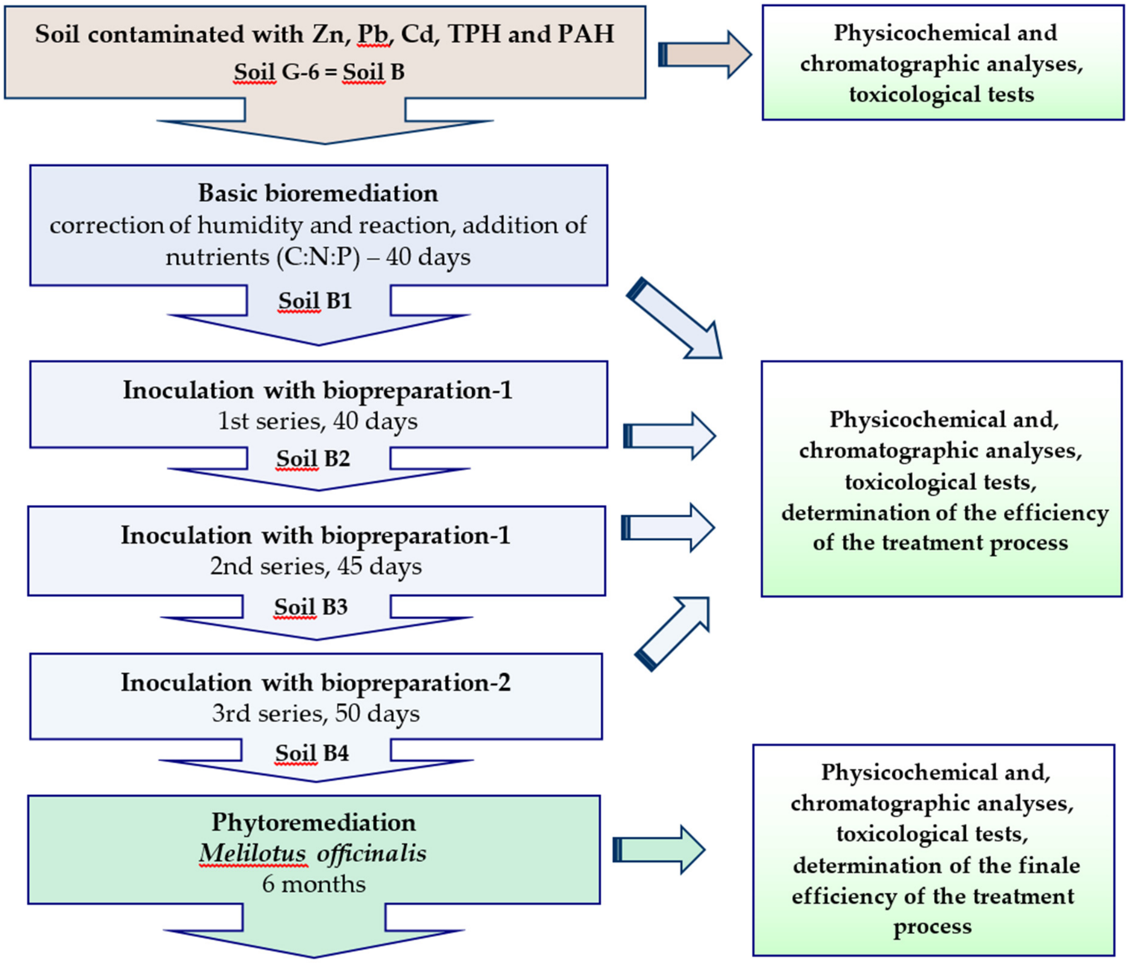

Figure S1. Schema of the purification of soil taken from G-6 waste pit (Soil B), led in semi-technical conditions (bioremediation – *ex situ* prism method, phytoremediation – pot tests).

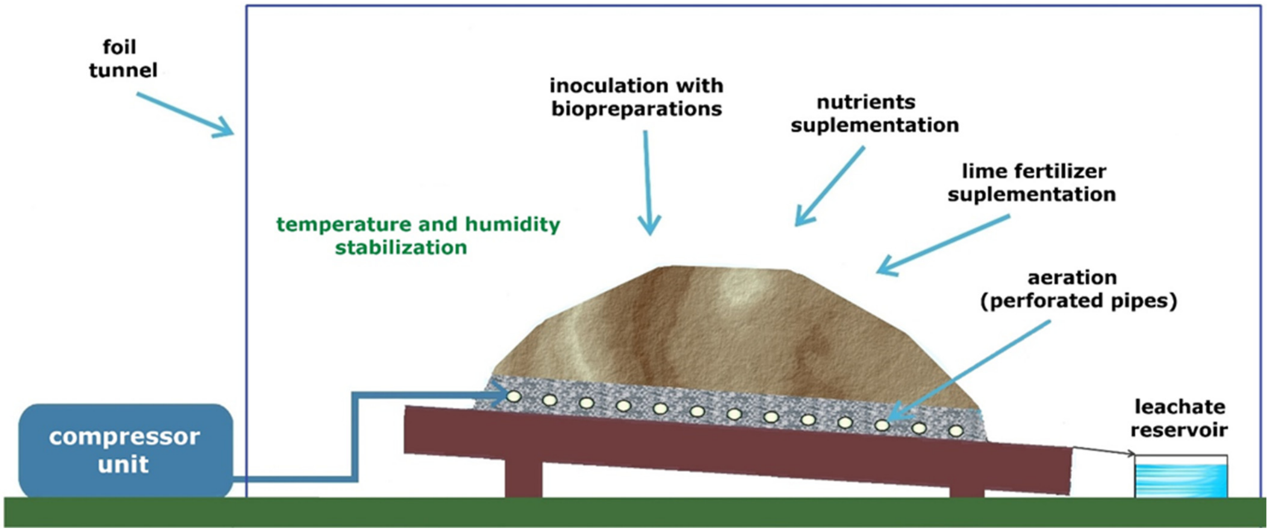

Figure S2. Scheme of the test stand for conducting the process of biodegradation of pollutants (TPH and PAH) in soil under semi-technical conditions (*ex situ* prism method).

Description of the experiment

Tests on the biodegradation process of TPH and PAH were carried out in real Soil B, contaminated with petroleum substances, in *ex-situ* semi-technical prism method. To protect the room against contamination an insulating foil was spread on the floor, on which a stand was placed, which design enabled drainage of water surplus and of liquid pollutants from the pile to the effluents tank. On the stand a layer of gravel bedding was placed, inside which a system of perforated pipes was situated, through which air from a compressor was pumped for proper aeration of soil during the biodegradation. Then a pile (approx. 50 kg) was formed from the soil polluted with aged transformer oil containing PCBs and it was covered with a foil tunnel, which allowed for maintaining a constant temperature inside the pile, ranging from 17 to 25°C. During the process of cleaning a constant humidity was maintained, within 20–25%, while the pH was stabilised at approx. 7.5–7.8, batching the fertilizer lime. Optimum proportions of biogenic substances N:P = 10:1 for the studied soil (Soil B1), were chosen by gradual proportioning of biogenic substances **(**nitrogen and phosphorus) in the form of 'Azofoska' mineral fertilizer. The process of inoculation was carried out by sprinkling the technological pile with biopreparation-1 (1st series 40 days – Soil B2, the 2nd series 45 days – Soil B3) and then biopreparation-2 from indigenous bacteria enriched in fungi and yeast (3rd series 50 days–Soil B4).

Table S1. Chemical and physical properties of the soil.

Parameter	Simple soil		
	**Soil A** *GPS: 22°4′42.69″ E 49°40′16.685″ N*	**Soil B** *GPS: 22°4′42.69″ E 49°40′16.685″ N*	**Soil B4**
pH $_{H_2O}$	6.1 ± *0.1*	6.3 ± *0.1*	6.5 ± *0.1*
Conductivity (*µS cm^{-1}*)	133 ± *8.8*	185 ± *9.0*	204 ± *9.2*
COD (*mg O$_2$ dm^{-3}*)	62 ± 5.0	224 ± *22.0*	162 ± *9,7*
Intial water moisture (%)	38.4 ± *3.3*	42.4 ± *5.5*	62.4 ± *5.6*
Chemical composition			
Cl^{-a}	17,7 ± *1.2*	278.0 ± *20.5*	306.5 ± *20.5*
S – SO_4^{2-a}	34.1 ± *29.0*	270.6 ± *19.2*	275.2 ± *19.2*
N – NH_4^{+a}	4.2 ± *0.4*	8.2 ± *1.9*	21.6 ± *1.9*
N – NO_3^{-a}	28.4 ± *2.5*	32.2 ± *2.0*	104.6 ± *7.1*
P– PO_4^{3-a}	6.5 ± *0.7*	18.3 ± *1.2*	21.4 ± *2.0*
Al_2O_3 [b]	450.2 ± *42.1*	749.8 ± *64.1*	749.1 ± *64.1*
SiO_2 [b]	488.1 ± *44.0*	185.5 ± *12.2*	187.3 ± *12.2*
Fe_2O_3 [b]	6.0± *0.6*	14.8 ± *2.2*	15.4 ± *2.2*
MgO [b]	3.4 ± *0.4*	8.0 ± *0.7*	9.1 ± *0.7*
CaO [b]	5.6 ± *0.2*	3.2 ± *0.3*	3.2 ± *0.3*
Sand (%)	44.2 ± *4.1*	27.0 ± *2.1*	27.5 ± *2.1*
Silt (%)	38.1 ± *3.6*	35.2 ± *3.1*	35.3 ± *3.1*
Clay (%)	17.7 ± *0.7*	37.8 ± *3.5*	37.2 ± *3.5*
Heavy metal content (*mg kg^{-1} dry mass*)			
As	1.8 ± *0.1*	10.0 ± *0.2*	12.0 ± *0.2*
Cd	1.5 ± *0.2*	11.0 ± *0.8*	9.5 ± *0.8*
Cr	26.8 ± *2.2*	49.3 ± *4.6*	47.2 ± *4.6*
Co	2,0 ± *0.2*	12,8 ± *0.2*	12,1 ± *0.2*
Cu	23.6 ± *2.3*	94.4 ± *4.0*	88.1 ± *3.7*
Pb	22.3 ± *2.4*	285.6 ± *22.0*	264.4 ± *22.0*
Mo	2.6 ± *0.3*	11.2 ± *0.3*	9.1 ± *0.3*
Ni	18.1 ± *1.2*	56.6 ± *4.8*	48.4 ± *4.5*
Sn	3.7 ± *0.4*	16.0 ± *0.4*	14.1 ± *0.4*
Zn	14.9 ± *1.5*	395.2 ± *26.4*	375.8 ± *26.4*
Hydrocarbons (*mg kg^{-1} dry mass*)			
C_6-C_{12}	66.2 ± *5.5*	5,481± *482*	218.7 ± *20.5*
C_{12}-C_{18}	135.4 ± *12.0*	26,842± *1.801*	328.6 ± *32.0*
C_{18}-C_{25}	108.5 ± *9.6*	11,884± *927*	383.7 ± *38.5*
C_{25}-C_{36}	42.2 ± *3.4*	4,032± *338*	566.6 ± *54.5*
TPH (*C_6-C_{36}*)	502.3 ± 42.4	56,371± *3,175*	1,931.9 ± *180.2*
Polycyclic aromatic hydrocarbons (*mg kg^{-1} dry mass*)			
Naphthalene (N)	0.18 ± *0.02*	81.80 ± *6.50*	13.56 ± *1.02*
Anthracene (A)	0.07 ± *0.01*	1.64 ± *0.08*	0.36 ± *0.06*
Chrysene (CH)	0.12 ± *0.02*	14.68 ± *0.84*	2.76 ± *0.32*
Benzo(a)anthracene (BaA)	0.11 ± *0.01*	22.16 ± *1.72*	4.56 ± *0.70*
Dibenzo(a,h)anthracene (DaA)	0.07 ± *0.01*	0.80 ± *0.05*	0.20 ± *0.03*
Benzo(a)pyrene (BaP)	0.06 ± *0.01*	1.24 ± *0.08*	0.34 ± *0.04*
Benzo(b)fluoranthene (BbF)	0.05 ± *0.01*	3.56 ± *0.29*	0.98 ± *0.15*
Benzo(k)fluoranthene (BkF)	0.03 ± *0.01*	1.00 ± *0.06*	0.28 ± *0.04*
Benzo(ghi)perylene (BghiP)	0.03 ± *0.01*	10.44 ± *0.92*	3.08 ± *0.48*
Indeno(1,2,3-cd)pyrene (IndP)	0.01 ± *0.01*	1.96 ± *0.10*	0.68 ± *0.12*
PAHs	0,73 ± *0.06*	139.28 ± *9.62*	26.80 ± *2.98*

COD) – chemical oxygen demand, [a]) content expressed as (mg kg^{-1} dry mass), [b]) content expressed as (g kg^{-1} dry mass), Soil A) soil taken from a forested area in the vicinity of the weathered drill wastes (correspond to control soil), Soil B) soil taken from G-6 weathered drill wastes, Soil B4)

soil B after bioremediation (basic bioremediation and inoculation with biopreparations prepared on the basis of nonpathogenic indigenous species of bacteria, fungi and yeasts).

Table S2. Species status of microorganisms strains included biopreparations.

Strain designation	Identification by classical methods	Identification by sequencing	% identity most similar sequence in GenBank	Safety category by ATCC
**Biopreparation-1**				
G-2	*Bacillus sp.*	*Bacillus subtilis*	99%/HE582781	1
G-4	*Burkholderia sp.*	*Burkholderia phenazinium*	99%/LC008479	1
G-5	*Gordonia sp.*	*Gordonia terrea*	99%/EU333873	1
G-7	*Mycobacterium sp.*	*Mycobacterium fredrikbergense*	99%/AF544630	1
G-9	*Mycobacterium sp.*	*Mycobacterium vanbaalenii*	99%/LN613105	1
G-10	*Pseudomonas sp.*	*Pseudomonas fluorescens*	99%/AY538263	1
G-12	*Pseudomonas sp.*	*Pseudomonas putida*	99%/KF278708	1
G-11	*Pseudomonas sp.*	*Pseudomonas rhodesiae*	98%/AB495138	1
G-13	*Rhodococcus sp.*	*Rhodococcus cercidiphylli*	100%/KT923346	1
G-14	*Rodococcus sp.*	*Rhodococcus erythropolis*	99% /CP007255	1
G-15	*Rhodococcus sp.*	*Rhodococcus opacus*	99% /CP009111	1
G-17	*Rhodococcus sp.*	*Rhodococcus ruber*	99%/EU168010	1
G-19	*Streptomyces sp.*	*Streptomyces aureus*	99%/AY094368	1
**Biopreparation-2 (Biopreparation-1 enriched with non-pathogenic species of fungi and yeast)**				
G_20	*Trichoderma sp.*	*Trichoderma asperellum*	99%EU077227	1
G-21	*Phanerochaete sp.*	*Phanerochaete chrysosporium*	98%/AF475147	1
G-22	*Candida sp.*	*Candida oleophia*	99%/HQ876036	1

Bacillus subtilis, Burkholderia phenazinium, Gordonia terrea, Mycobacterium fredrikbergense, Mycobacterium vanbaalenii, Pseudomonas fluorescens, Pseudomonas putida, Pseudomonas rhodesiae, Rhodococcus cercidiphylli, Rhodococcus erythropolis, Rhodococcus opacus, Rhodococcus ruber, Streptomyces aureus, Trichoderma asperellum, Phanerochaete chrysosporium, Candida oleophia

Table S3. Indicators of n-alkane biodegradation degree after consecutive purification stages of waste from soil B (*ex-situ* method)

Biodegradation index	Soil B	Soil B1	Soil B2	Soil B3	Soli B4
n-C_{17}/Pr	15.075 ± 1.257	8.075 ± 0.621	2.444 ± 0.185	0.539 ± 0.058	0.097 ± 0.009
n-C_{18}/F	8.771 ± 0.868	3.983 ± 0.283	1.522 ± 0.085	0.568 ± 0.044	0.126 ± 0.0113

Table S4. Equation coefficients of mathematical model biodegradation of TPH and PAH in consecutive stages of soil B treatment (laboratory conditions, *ex-situ* method)

Soil after the next stages of cleaning	TPH		∑ n-C_8–n-C_{22}		∑ n-C_{23}–n-C_{36}		PAH	
	k [d^{-1}]	r^2	k [d^{-1}]	r^2	k [d^{-1}]	r^2	k [d^{-1}]	r^2
Soil B1	0.0089 ± 0.003	0.9865	0.0099 ± 0.006	0.9952	0.0032 ± 0.002	0.9572	0.0048 ± 0.002	0.9818
Soil B3	0.0179 ± 0.012	0.9904	0.0227 ± 0.012	0.9789	0,0095 ±0.006	0.9584	0.0110 ± 0.011	0.9623
Soll B4	0.0196 ± 0.014	0.9975	0.0251 ± 0.017	0.9879	0.0122 ± 0.011	0.9741	0.0141 ± 0,016	0.9842

Microbiological analysis

Isolation of G-6 pit soil was done on a mineral substratum with addition of 15g of agar and crude oil. There were used 10 probations (minimum 100g each), taken in various sites of each waste pit, in order to obtain huge diversity of microorganisms species able to petroleum hydrocarbons degradation. Next, a standard streak technique was done (3-7 times) to obtain pure strains. The aim of the research was to analyse basic qualities such as: motility, growth in oxygen free/ oxygen conditions (at temperatures from 4°C to 40°C in a wide range of pH), NaCl concentration tolerance to 10% and ability to use crude oil and diverse hydrocarbons (heptane, n-decane, n-dodecane, n-octadecane, n-nonadecane, n-docosane, n-hexacosane, phenol, toluene, xylene and naphthalene) as a sole carbon source. In a case of volatile hydrocarbons and crude oil, incubation was led in desiccator in their atmosphere. Liquid hydrocarbons were added directly to the medium, whereas solid hydrocarbons were added directly to the liquid medium or agar plates were covered with them with application of sublimation method. Microorganisms with ability to degradation of both aliphatic and aromatic hydrocarbons or with a wide range utilization of aliphatic hydrocarbons were selected. Another selection criterium was rate of biofilm formation between two phases (oil and water) when n-hexadecane was the only carbon source in the mineral medium. The test was done in 100 ml flasks containing 75ml of the medium and 5ml of n-hexadecane. It enabled estimation of rate of microorganisms metabolism adaptation to the utilization of hydrocarbons. Identification of microorganisms was led with application of both classical and molecular methods. Classical methods consisted of: estimation of microorganisms morphology (shape and colour of colonies grown on the agar plates, mycelia of fungi and pseudomycelia of actinobacteria), microscope techniques (cell morphology, conidiophores and conides, motility, Gram, acid-fast, capsule and spore staining). In addition, the methods included: biochemical properties and the use of selective media. There were applied available biochemical tests and an automatic system of Mini API made by Biomerieux (tests to automatic identification: ID 32GN, ID 32STAPH and to manual identification: API Coryne and API 50CHB).

In order to analyse sequencing of DNA coding 16S rRNA of bacteria and 18S rRNA of fungi, a DNA gene was isolated according to modified Marmur's Method, which uses extraction with a solution of phenol/chloroform/isoamyl alcohol (volume ratio – 25:24:1) Then, there was applied a method of enzymatic amplification (PCR-I – polymerase chain reaction) of DNA fragments, with the use of starters flanking about a 500-nucleotide fragment of a gene and length of DNA fragments on agarose gel was measured. The next step was an enzymatic reaction of sequencing (PCR-II) with application of a sequencing kit (BigDye Terminator v 3.0 Ready Action Cycle Sequencing Kit) made by Amersham Biosystems. The analysis of sequencing was done with the use of a capillary sequencer of ABI Prizm 3100 Genetic Analyser. Identification was possible due to MicroSeq - a data base of 16S rDNA sequences and a BLAST programme on a website of National Center of Biotechnology Information [http://www.ncbi.nlm.nih.gov/BLAST].
